# Not so clonal asexuals: Unraveling the secret sex life of *Artemia parthenogenetica*

**DOI:** 10.1002/evl3.216

**Published:** 2021-02-08

**Authors:** Loreleï Boyer, Roula Jabbour‐Zahab, Marta Mosna, Christoph R. Haag, Thomas Lenormand

**Affiliations:** ^1^ CEFE Univ Montpellier, CNRS, Univ Paul Valéry Montpellier 3, EPHE, IRD Montpellier France

**Keywords:** Artemia, asexuals, automixis, contagious asexuality, rare sex, recombination

## Abstract

The maintenance of sex is paradoxical as sexual species pay the “twofold cost of males” and should thus quickly be replaced by asexual mutants reproducing clonally. However, asexuals may not be strictly clonal and engage in “cryptic sex,” challenging this simple scenario. We study the cryptic sex life of the brine shrimp *Artemia parthenogenetica*, which has once been termed an “ancient asexual” and where no genetic differences have ever been observed between parents and offspring. This asexual species rarely produces males, which can hybridize with sexual females of closely related species and transmit asexuality to their offspring. Using such hybrids, we show that recombination occurs in asexual lineages, causing loss‐of‐heterozygosity and parent‐offspring differences. These differences cannot generally be observed in field‐sampled asexuals because once heterozygosity is lost, subsequent recombination leaves no footprint. Furthermore, using extensive paternity tests, we show that hybrid females can reproduce both sexually and asexually, and transmit asexuality to both sexually and asexually produced offspring in a dominant fashion. Finally, we show that, contrary to previous reports, field‐sampled asexual females also rarely reproduce sexually (rate ∼2‰). Overall, most previously known facts about *Artemia* asexuality turned out to be erroneous. More generally, our findings suggest that the evidence for strictly clonal reproduction of asexual species needs to be reconsidered, and that rare sex and consequences of nonclonal asexuality, such as gene flow within asexuals, need to be more widely taken into account in more realistic models for the maintenance of sex and the persistence of asexual lineages.

**Impact summary**

Although supposedly advantageous, asexual reproduction is rare in nature, compared to sexual reproduction. Most models explaining the maintenance of sex, “the queen of problems” in evolutionary biology for decades, include a sex‐asex contrast. In the vast majority of models, asexuals are simplified as obligate and clonal, where the maternal genome is transmitted faithfully (barring new mutations). Even though other asexual reproductive modes exist and their population genetic consequences are starting to be understood, moving beyond this simplification is extremely challenging theoretically and in practice. In this article, we focused on a well‐known asexual taxon, *Artemia parthenogenetica*, whose reproductive mode has been studied for over a century. We challenged supposedly established facts about its asexuality through experiments using “rare males,” which are sometimes produced in asexual lineages. They were used to produce sex‐asex crosses and backcrosses with a closely related sexual species. This allowed us to unravel characteristics that profoundly change the view on almost all aspects of *Artemia* asexuality: We show that these supposedly “obligate asexuals” can sometimes reproduce sexually and that asexuality can be sexually transmitted via both males and females. We also show that recombination was selected against, but not totally lost in asexual *Artemia*. These findings explain how asexuals can appear as clones, despite not being clonal. It appears reasonable to think that similar discoveries could be made in other asexual taxa through detailed investigations using nonstandard approaches. This is crucial, as evolutionary processes, including the advantage compared to sex, may strongly differ between clonal and nonclonal asexuals. Taken together, our study shows that real asexuals are far from the caricature used in current models. If accounted for, this could help understanding the maintenance of sex with a more comprehensive view of reproductive mode diversity.

The prevalence of sexual over asexual reproduction in eukaryotes is generally explained by the fact that, even though the costs of sex are high (including the famous putative twofold cost of males, Maynard Smith 1978, but see Meirmans et al. 2012), the costs of asexuality, particularly when clonal, are even higher (e.g., increased accumulation of deleterious mutations, slower rate of adaptation, Otto and Lenormand 2002; Otto 2009; Schön et al. 2009). However, clonality—the production of offspring genetically identical to their mothers, barring new mutations—may not be as ubiquitous as expected (Engelstädter 2008; Archetti 2010; Lenormand et al. 2016). In particular, rare events of recombination and sex in asexuals might be missed because they are difficult to detect. These occurrences of rare sex in “asexuals” may be especially frequent in young asexual lineages emerging within a population of sexual ancestors (i.e., when their relative fitness matters most). This would lead to an underestimation of nonclonal asexuality in nature and undermine the classical “paradox of sex” scenario, which considers that asexuality emerges within sexual species through strictly clonal mutants.

In this article, we study recombination and sexual reproduction in *Artemia parthenogenetica*, which was once described as an “ancient asexual” (Judson and Normark 1996). *Artemia parthenogenetica* is a heterogeneous group of brine shrimps encompassing diploid and polyploid asexuals. Here, we focus on diploids (hereafter *Ap2n*), whose reproductive mode has been debated throughout the 20th century (Barigozzi 1944; Narbel‐Hofstetter 1964; White 1973; Cuellar 1987; Neiman et al. 2009). Recent genetic data suggest that they reproduce by “central fusion” automixis (Nougué et al. 2015). This type of automixis can correspond, at the cellular level, either to the fusion of meiotic products separated at meiosis I or to the abortion of meiosis I (sometimes called meiotic apomixis; Archetti 2010). Both cases have the same genetic consequences and maintain diploidy without fertilization (Asher 1970). With central fusion automixis, centromeric regions maintain maternal heterozygosity (i.e., are transmitted clonally), but if there is recombination, centromere‐distant regions can become autozygous, that is, undergo loss of heterozygosity (hereafter, LOH; Stenberg and Saura 2009; Svendsen et al. 2015). Note that this expectation applies only to species with monocentric chromosomes, as is the case in *Artemia* (Yarmohammadi and Pourkazemi 2004). This reproductive mode was inferred indirectly from population genetic data (strongly contrasting *F*_IS_ levels among different markers in wild populations; Nougué et al. 2015). However, no genetic variation has ever been observed within isofemale lines of *Ap2n*. Browne and Hoopes (1990) found no change in allozyme genotypes in three heterozygous lines maintained for three years in the laboratory. Similarly, Nougué et al. (2015) found no genotypic changes at five heterozygous microsatellite loci in three isofemale lines maintained for 20–37 months. This has been considered as evidence for essentially clonal reproduction (or central fusion automixis without recombination, which is genetically equivalent; Abreu‐Grobois 1983; Abreu‐Grobois and Beardmore 2001). Yet this conclusion does not account for potential difficulties in detecting nonclonal reproduction: Indeed, centromere‐distant loci that frequently recombine with the centromere and therefore have high rates of LOH may already have lost heterozygosity and hence no further LOH can be detected. In other words, centromere‐distant loci are expected to be mostly homozygous (and have positive *F*_IS_; Nougué et al. 2015), except for short periods of times following the occurrence of a new mutations (Engelstädter 2008). In contrast, loci close to the centromere may only rarely experience LOH and therefore have high heterozygosity (and negative *F*_IS_; Nougué et al., 2015). In principle, the recombination and new LOH events could be detected at these loci. However, if LOH rate is low, they will, by definition, only rarely be observed. Hence, detecting recombination in automicts is methodologically challenging, irrespectively of the genomic location, with high or low LOH rates. This in turn suggests that erroneous inferences of clonality could easily occur.

In addition to this issue of recombination, *Ap2n* lineages are known for their production of “rare males” by parthenogenesis (0–1.7% of all offspring; Browne & Hoopes, 1990; Maccari et al., 2013). The contribution of these males to *Ap2n* reproduction remains unresolved. Their ability to cross with sexual females and transmit asexuality to their offspring is termed “contagious asexuality” and could in principle generate a large diversity of new asexual lineages. This mechanism has been demonstrated in a handful of asexual species that produce rare males (Aphids: Jaquiéry et al., 2014; *Daphnia*: Paland et al., 2005; Parasitoid wasps: Sandrock & Vorburger, 2011). Asexual hermaphrodites can also sexually transmit asexuality through their male function (Van Dijk, 2009). This happens in some animals (D'Souza et al., 2004) and potentially in many plants (Hörandl & Paun, 2007). However, the overall prevalence of contagious asexuality among extant asexuals is difficult to establish, as it requires the identification of rare males (or male function), successful crosses with closely related sexuals, and the assessment of the reproductive mode of sex‐asex hybrids. The first studies on contagious asexuality in *Ap2n* found no evidence for transmission of asexuality after hybridization with closely related sexuals (Bowen et al., 1978). A more recent study showed that rare *Ap2n* males can transmit asexuality, but concluded that transmission is recessive (Maccari et al., 2014). Recessivity would strongly limit the appearance of new asexual lineages, as no asexuals are generated in the F1. New asexual lineages may only occur after another rare cross, mating between rare F1 or a backcross with another rare, asexually derived male. Furthermore, although *Ap2n* populations are widely distributed, the geographical distribution of sexual species is narrow; however, they do currently overlap in central Asia in few locations (Browne & MacDonald, 1982; Agh et al., 2007; Muñoz et al., 2010). The relevance of contagious asexuality in this species is therefore unclear, and, given the divergent results of earlier studies, some doubts also remain regarding the mode of inheritance of asexuality.

The purpose of the present study was to revisit the reproductive biology of a supposedly well‐known obligate asexual and to use this example to showcase the correspondence between real asexual species and their caricatures used in most models. In particular, we reconsidered features of *A. parthenogenetica* reproduction through a series of five critical experiments. First, we tested whether recombination occurs in *Ap2n*. To do so, we used contagious asexuality to experimentally generate new hybrid asexual lineages. Note that hybridization itself can sometimes induce asexuality (“balance hypothesis”; Moritz et al., 1989). However, in *Artemia*, experimental hybrid crosses between sexual species never resulted in asexual offspring (Clark & Bowen, 1976; Pilla & Beardmore, 1994; Abatzopoulos et al., 2002; Maccari et al., 2013), and several further lines of evidence indicate that the “balance hypothesis” was unlikely to operate in our crosses (see discussion). We crossed *Ap2n* “rare males” with females from the closest sexual species (*A. sp. Kazakhstan*, hereafter *Akaz*; Muñoz et al., 2010). Because of hybridity, these lineages are expected to show high heterozygosity, which is expected to greatly improve the likelihood to detect LOH events, if they occur at all. Second, we investigated the reproductive mode of these F1 sex‐asex hybrid females by pairing them with *Akaz* males and subjecting the resulting offspring to paternity tests. Third, we asked whether contagious asexuality occurs only via “rare males” or may also happen through females carrying asexuality genes, while, at least partially, retaining sexual function. We assessed this possibility by crossing laboratory‐produced F1 sex‐asex hybrid females with *Akaz* males and testing whether some of the resulting offspring females were able to reproduce asexually. Fourth, we tested whether females from relatively older, field‐sampled *Ap2n* asexual lineages can sometimes reproduce sexually (they were hitherto thought to be 100% obligate asexuals). To answer this question, we conducted mass‐cross experiments combined with paternity tests. Finally, we asked whether recombination had evolved in asexual lineages. To this end, we used the proportion of males produced during asexual reproduction (i.e., the frequency of rare males) as a proxy for recombination rate in *Ap2n* asexuals, F1 sex‐asex hybrids, and several generations of backcrosses to *Akaz*. Overall, the results of these five experiments entirely change our view of *Artemia parthenogenetica* asexuality. Similar experimentation could lead to reappraisal in other systems, and our results highlight that models on the maintenance of sex may require to be updated, in particular by including more realistic assumptions about asexuality, beyond strict clonality.

## Methods

In the different experiments, we used standard raising conditions for *Artemia*, as described in Lievens et al. (2018). Details are provided in Supporting Information part 1.

### EXPERIMENT 1: LOH IN SEX‐ASEX HYBRIDS

To investigate LOH, we generated hybrid lineages via contagious asexuality, crossing rare males from two *Ap2n* lineages, Aigues‐Mortes (France), hereafter *P1*, and Urmia (Iran), hereafter *P2* (see Supporting Information part 1), with *Akaz* females. Asexual offspring were isolated and propagated asexually in 34 lineages for up to 13 generations. To screen for LOH, we genotyped last‐generation individuals for seven microsatellite markers that were heterozygous in the F1. Events of LOH were then traced back to the generation in which they occurred (Supporting Information part 2, Fig. S1). We included five informative loci in the data that were analyzed using likelihood models in Mathematica version 9.0 (Wolfram Research, 2012), investigating effects of the cross (*P1×Akaz* or *P2×Akaz*), time (i.e., generation number at which LOH happened), and locus (Table S1).

### EXPERIMENT 2: REPRODUCTIVE MODE OF F1 HYBRID FEMALES

The second experiment was aimed at identifying the reproductive mode of females produced by contagious asexuality. We used a previously established protocol (Maccari et al., 2014) with some modifications. We crossed a rare male from *P1* and a rare male from *P2* with *Akaz* females, isolated all F1 hybrids, and identified the sex of the offspring. During period 1, F1 females were kept isolated for 14 (*P2×Akaz*) or 30 days (*P1×Akaz*). During a period 2, we paired them with an *Akaz* male. A longer period 1 was applied to the *P1×Akaz* cross, which was performed after *P2×Akaz*, to increase the chance to observe asexual reproduction during isolation in this second cross. Period 1 was only used as a check that females could reproduce in isolation. Offspring produced during period 2 were genetically tested using microsatellites to determine whether they were produced sexually or asexually (Supporting Information part 3, Table S2). The proportion of sexually versus asexually produced offspring was estimated only using offspring produced during period 2. Females that only produced asexual clutches during both periods were labeled as “asexual,” those that only produced sexual clutches when paired and no clutches in isolation as “sexual.” It is, however, possible that females capable of both modes of reproduction only displayed one reproductive mode during the experiment because of the limited number of clutches and offspring tested. To account for this uncertainty, data were analyzed using likelihood models in Mathematica version 9.0 (Wolfram Research, 2012), distinguishing the different categories of females, and testing the effects of the origin of the cross (*P1×Akaz* or *P2×Akaz*) on the reproductive mode (Table S3).

### EXPERIMENT 3: CONTAGIOUS ASEXUALITY VIA HYBRID F1 FEMALES

The third experiment was designed to detect whether asexuality could be transmitted sexually by females (contagious asexuality through females). We used 12 clutches produced by the paired F1 females of *P1×Akaz* from experiment 2. Five of these were produced sexually, as verified by paternity testing. From each clutch, we isolated one to six female offspring for four weeks and recorded whether they were able to reproduce asexually (Supporting Information part 4, Table S4). The occurrence of such asexual female offspring would show that asexuality could be sexually and maternally transmitted, thus demonstrating contagious asexuality via females.

### EXPERIMENT 4: RARE SEX IN *Ap2n* FEMALES

In the fourth experiment, we investigated whether rare sex could occur in field‐sampled *Ap2n* females of the two populations studied in this article. We placed 115 *P1* females with 57 *Akaz* males and 52 *P2* females with 25 *Akaz* males in large tanks. We used *Akaz* males for practical reasons (easy availability of males with diagnostic loci) and because *Akaz* is the closest related sexual species to *Ap2n* (Muñoz et al., 2010). Eight and four male offspring were obtained among 1828 and 1061 offspring, respectively (Table S5). Any male offspring appearing in these tanks were therefore either produced asexually by *Ap2n* females (i.e., rare males) or produced sexually by hybridization between the *Ap2n* females and the *Akaz* males. We used paternity tests on all these male offspring to determine whether they were produced by sexual reproduction (Supporting Information part 5).

### EXPERIMENT 5: ESTIMATING RECOMBINATION RATE IN AUTOMICTS

The fifth experiment was designed to assess whether recombination rate had evolved in *Ap2n* lineages, compared to their sexual *Akaz* ancestor. One of the few hypotheses explaining how rare males may be produced in *Ap2n* suggests that they result from LOH at the sex‐determining locus during oogenesis (Stefani, 1964; MacDonald & Browne, 1987; Browne & Hoopes, 1990; Abreu‐Grobois & Beardmore, 2001). Females are ZW (Bowen, 1963; Stefani, 1963; de Vos et al., 2013), thus LOH could result in ZZ or WW offspring, WW potentially being nonviable and ZZ being rare males. According to this, the rate of rare male production in a lineage would be a proxy for the recombination rate between the centromere and the sex locus in this lineage (Browne & Hoopes, 1990). This hypothesis has not been experimentally addressed to date, but is consistent with our observations (see Discussion). We used the rate of rare male production as a proxy for automictic recombination rate to investigate how recombination evolves in automictic lineages. We predicted that, because it leads to LOH that may expose deleterious recessive mutations, recombination may be selected against in automictic lineages. Compared to asexuals, F1 hybrids and further backcross generations to *Akaz* sexuals should thus show increased recombination rates, reaching saturation once backcrosses have integrated all recombination‐controlling genes of the *Akaz* sexual species. We used a *P3×Akaz* cross using a rare male from another Aigues‐Mortes population (*P3*), and an *Akaz* female. We then used repeated backcrosses on *Akaz* to introgress the asexuality genes of *Ap2n* into an increasingly *Akaz* (and thus sexually derived) genome. We maintained asexuality by selecting each generation males whose daughters were able to reproduce asexually. We recorded the rate of rare male production (denoted α) in asexually produced clutches from the F1 for up to four backcross generations (Table S6). Data were analyzed using likelihood models with Mathematica version 9.0 (Wolfram Research, 2012). The models investigated how mean α changed throughout subsequent generations of crossing and back‐crossing (in a linear, quadratic, or step‐wise fashion). The variance of α among lineages was also fitted either assuming that recombination rate was controlled by a major gene (monogenic models, where we expect two categories of females in the backcrosses) or that is was polygenic (polygenic models, where we expect a continuous distribution of recombination rates among females; Supporting Information part 6, Table S7).

## Results

### EXPERIMENT 1: LOH IN SEX‐ASEX HYBRIDS

**Figure 1:**
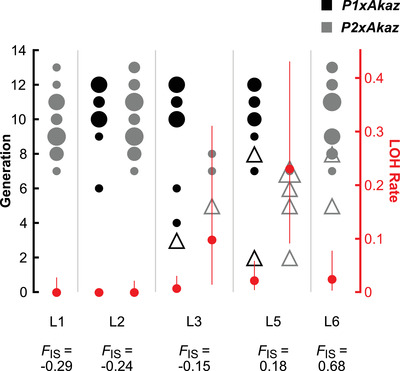
Loss and retention of heterozygosity at six microsatellite loci (L1 to L6) in asexual F1 hybrid lineages in experiment 1. Disks represent *P1×Akaz* (black) and *P2×Akaz* (gray) hybrid lineages that retained heterozygosity, and triangles lineages that lost heterozygosity. The generation at which heterozygosity was lost or, alternatively, the generation until which the lineage was followed without LOH is indicated for each disk and triangle on the left axis. The size of the symbols represents the number of lineages with the same value. Small and large triangles correspond to 1 or 2 lineages, respectively. Red dots and bars represent per‐locus LOH rates and support limits (right axis) estimated from our best model. For each locus, the *F*_IS_ found in natural populations by Nougué et al. (2015) is indicated below. Note that L1 and L6 were not initially heterozygous in *P1* lineages and are therefore not represented. Note also that the representation of the generation in the figure does not account for partial nonindependence of some of the lineages due to sharing part of their ancestry (see Fig. S1 for the exact pedigree and LOH events of all lineages). The model estimates are, however, not affected by this as they account for partial nonindependence.

Microsatellite analysis of the hybrid *Ap2n* lineages showed that five out of seven loci (including the two loci later discarded from the statistical analysis; see Supporting Information part 2) that were initially heterozygous in F1 underwent LOH in at least one out of 34 asexual hybrid lineages within 1–13 generations (Fig. S1). Moreover, according to our best model (ΔAICc = 2.2; Table S1), LOH rates varied between the two populations of origin and among loci, with LOH occurring mainly at loci that show excess homozygosity (compared to Hardy‐Weinberg proportions) in natural *Ap2n* populations (Fig. 1). With central fusion automixis, we expect such heterogeneity among loci, depending on their chromosomal position. Centromere‐distal loci should lose heterozygosity and show heterozygosity deficit in natural populations, whereas loci close to the centromere should show the reverse pattern (Nougué et al., 2015; Svendsen et al., 2015). The close correspondence between LOH observed in our laboratory F1 crosses and heterozygosity patterns independently observed in asexuals sampled in the field indicate that our crosses qualitatively reflect recombination actually occurring in natural *Ap2n*.

### EXPERIMENT 2: REPRODUCTIVE MODE OF F1 HYBRID FEMALES

**Figure 2:**
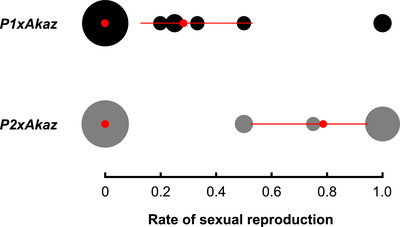
**Frequency of sexual clutches in F1 hybrids in experiment 2**. Disks represent *P1×Akaz* (black) and *P2×Akaz* (gray) F1 females, with the size of symbols proportional to the number of females with the same value. Red dots and bars represent the rate of sexual reproduction and support limits as estimated from our best model. The estimated proportion of females in the two categories (fully asexual vs. mixed) does not significantly differ between the two crosses and is estimated at 56 % asexual (support limits: 35–71%) and 44% mixed (support limits: 28–66%).

In contrast to previous findings (Maccari et al., 2014), our crosses revealed that a large fraction of virgin F1 females were able to reproduce while isolated (i.e., asexual reproduction during period 1): 89% ± 5% (SE) of the *P1×Akaz* hybrids and 45% ± 5% (SE) of the *P2×Akaz* hybrids (Table S2). The different proportions between *P1×Akaz* and *P2×Akaz* hybrids may be explained by different duration of period 1 (see Methods and Discussion). When paired with an *Akaz* male, some females continued reproducing asexually, as verified by paternity testing (Table S2). Indeed, 66.1%  ± 6% (SE) of females whose reproductive mode could be identified by paternity testing only ever produced asexual clutches throughout their lives (although the maximum number of clutches observed for a given female was five). Yet 25.4% ± 6% (SE) of females showed “mixed” reproduction, that is, they produced both asexual and sexual clutches (Table S2). Note that, within a given clutch, all offspring were produced by the same reproductive mode (Supporting Information part 3). There was only limited evidence for the existence of females with pure sexual reproduction: Among the 18 (of a total of 59) females that did not reproduce while isolated and whose reproductive mode could be identified by paternity testing, only five (all from the *P2×Akaz* cross) produced only sexual clutches when paired with a male (Table S2). However, each of them produced only one or two clutches, so that it is difficult to exclude that they would have been able to reproduce asexually in subsequent clutches. Accordingly, our most likely statistical model (Table S3) did not support the occurrence of purely sexual females (ΔAICc = 2.1) and included only two categories of F1 females (Fig. 2): 56% purely asexual and 44% mixed (not significantly different from 50% each, ΔAICc = 0.4 with a model where the proportion was fixed to 50%). Among the mixed F1 females, those from the *P2×Akaz* cross produced significantly (ΔAICc = 7.3 with a model where there is no cross effect) more sexual clutches than those from the *P1×Akaz* cross (79% vs. 29%; Table S3). The second‐best model (ΔAICc = 1.1) also included two categories of females but mixed females had slightly heterogeneous rates of sexual reproduction, which differed between *P1×Akaz* and *P2×Akaz* crosses.

### EXPERIMENT 3: CONTAGIOUS ASEXUALITY VIA HYBRID F1 FEMALES

The third experiment showed that contagious asexuality, which previously was thought to happen only via rare males, can also occur via females. Indeed, we found that asexual “mixed” females, when crossed with a sexual male, could transmit asexuality to some of their sexually produced daughters. Among the 12 clutches produced by *P1×Akaz* females paired with *Akaz* males, five were later identified as being the result of a sexual cross (i.e., being a first‐generation backcross), whereas the others were found to be asexually produced. A total of 10 virgin female offspring from these five clutches were isolated. Two of them successfully reproduced in absence of males (production of cysts; Table S4), showing that they were capable of asexual reproduction.

### EXPERIMENT 4: RARE SEX IN *Ap2n* FEMALES

The fourth experiment showed that *Ap2n* females engage in rare sexual reproduction. The mass‐cross between *P1* females and *Akaz* males produced 1828 offspring, of which eight were males. Paternity tests revealed that four of these were rare males, produced asexually, whereas four were *P1×Akaz* hybrids. The hybrids were possibly the result of a single copulation between an *Akaz* male and a *P1* female (in experiment 2, we found that, in the clutches produced by F1 hybrids, all offspring were produced by the same reproductive mode; Supporting Information part 3). They were found at the same time, likely had the same age, and, according to their genotype, it is possible that they had the same father (Table S5). Fertilization may either have resulted in diploid or triploid offspring, depending on whether the mother produced diploid or haploid ovules. To distinguish between these two hypotheses, we checked whether the sexually produced males inherited both maternal alleles at loci that were heterozygous in *P1* females. In three male offspring that could conclusively be tested (one was inconclusive due to shared null‐alleles), the male inherited only one of the two female alleles (Table S5). It is therefore likely that the females from the *P1* population can (rarely) undergo normal meiosis and produce haploid gametes. The alternative explanation of fertilization of a diploid egg that underwent LOH is unlikely, as the estimated LOH rates at the same loci in experiment 1 are 0.021 and 0.025 per generation (L5 in *P1×Akaz* F1 and L6 in *P2×Akaz* F1, respectively; Fig. 1). In the second mass cross (involving *P2*), we found four males among 1061 offspring, but they were all rare *Ap2n* males.

### EXPERIMENT 5: ESTIMATING RECOMBINATION RATE IN AUTOMICTS

**Figure 3:**
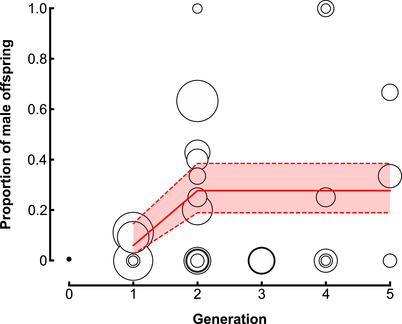
**Proportion of asexually produced males in different *P3*** *×* ***Akaz* backcross generations in experiment 5**. Empty disks represent sex ratios among asexually produced offspring per female, with the area of the circle being proportional to the number of offspring. The thick red line shows the mean proportion of male offspring as estimated from our best model (reaching a plateau at 27%), and the dashed red lines represent the confidence interval of this mean. The black dot at generation zero indicates the proportion of rare male production reported for *P3* (3.93‰; Maccari et al., 2013).

We found that α, the rate of rare male production, was higher in F1 hybrids than in asexual populations (Fig. 3; Maccari et al., 2013). The model best fitting the data was a polygenic model with a step variation of the mean α, and a quadratic effect on the variance of α (Table S7). It was better than the best monogenic model (ΔQAIC = 6.3). This model shows that α further increased in the first backcross generation, but not significantly afterward, plateauing at a value of 27% (Fig. 3). The variance of α was found null among F1, increased in subsequent backcross, and returned to zero in the fourth backcross generation (Fig. S2). This is expected under a polygenic control of recombination, with variation introduced by the introgression of *Akaz* recombination genes until all *Ap2n* recombination genes are replaced by *Akaz* ones during successive backcrosses. The second‐best model (ΔQAIC = 1.8) was similar but mean α followed a cubic variation with a qualitatively similar shape.

## Discussion

We show that contagious asexuality can be used to generate hybrids and backcrosses and that this experimental approach provides excellent opportunities to investigate, in detail, the reproduction biology of asexuals, the inheritance of asexuality, as well as the mechanisms and genetic consequences of asexuality. Our results suggest that asexuality in hybrids is transmitted from the asexual parent, rather than caused by hybridization itself. In experiment 1, loci with a high LOH rate in hybrids tend to have high *F*_IS_ (homozygosity excess) in wild *Ap2n* (see Fig. 1 and Nougué et al., 2015), which indicates that the same type of asexuality (central fusion automixis) occurs in hybrids and their asexual parents (this correspondence is expected if asexuality is inherited but very unlikely if asexuality is caused by hybridization). Furthermore, in experiment 5, it was possible to maintain asexuality (by selecting males that were likely to carry asexuality genes) during up to four backcross generations. About 97% of the genome of these fourth‐generation backcross individuals is of *Akaz* origin. This supports our assumption that asexuality is passed on from *Ap2n* to these individuals by the transmission of a small part of the genome that carries asexuality gene(s) rather than by hybridization per se, thus arguing against the balance hypothesis as a plausible explanation for our results. We found five major results, corresponding to the five experiments reported in this article. Each of these experiments revealed surprises, which were entirely new to *Artemia* biology (summarized in Table S8) and which have major implications for the evolution of asexuality, as they suggest that similar hidden features of asexuality could be found in many other asexual taxa if investigated in sufficient detail.

### EVIDENCE FOR CENTRAL FUSION AUTOMIXIS IN *Ap2n*

We provide the first demonstration of genetic differences between parents and their asexually produced offspring in *Artemia*. The estimated per‐locus LOH rates (up to 23.0% per generation) are much higher than typical gene conversion rates (Liu et al., 2018), and LOH therefore likely results from recombination. The direct observation of partial LOH (“partial” because only observed for some loci) provides strong evidence in favor of central fusion automixis in *Ap2n*. As the *Artemia* example shows, a lack of parent‐offspring differences does not necessarily prove clonality, although it is frequently interpreted as such (Stenberg & Saura, 2009; Dukić et al., 2019). Indeed, central fusion automixis is genetically equivalent to clonality only in the complete absence of recombination. If recombination occurs, it has different genetic consequences (Engelstädter, 2017). Yet even in the presence of recombination, parent‐offspring differences may remain unnoticed because, like in *Artemia*, and depending on genomic location, there is either no heterozygosity to lose (regions with high recombination likely have lost heterozygosity before) or there is a low chance to observe it (regions with low recombination can be heterozygous, but they are unlikely to undergo LOH). Indeed, no genetic differences were found in field‐sampled *Ap2n* lineages across tens of generations (Browne & Hoopes, 1990; Nougué et al., 2015). Our approach to generate new asexual hybrids was key for the detection of LOH. The same or similar approaches could be used in other systems with rare males or in systems where crosses are possible by other means (e.g., partial asexuals, hermaphrodites).

### RECOMBINATION RATE MAY BE SELECTED AGAINST IN AUTOMICTS

Recombination in automictic asexuals has different consequences compared to recombination in sexuals. Especially in newly formed asexuals, LOH exposes recessive deleterious mutations, leading to a phenomenon similar to inbreeding depression (loss‐of‐complementation; Archetti, 2010). This may explain the low rate of sex‐to‐asex transitions in eukaryotes with only few lineages being able to escape this early fitness decrease (Archetti, 2010; Engelstädter, 2017). Escaping LOH may also be achieved by bypassing meiosis altogether, but this is likely to pose other severe problems (e.g., perturb epigenetic reset, Lenormand et al., 2016; or other problems, Engelstädter 2008). Another possible way to avoid the deleterious consequences of LOH is to reduce the recombination rate, which may explain why many extant asexuals genetically behave like “clones” (Goudie et al., 2012; Engelstädter, 2017; Dukić et al., 2019). Our results support this hypothesis, as we show that (a) hybrids from the *P2×Akaz* lineage have substantially higher LOH rates than hybrids from the *P1×Akaz* lineage (Fig. 1), suggesting that recombination rate can vary and thus evolve in automictic lineages; (b) we estimated an increased rare male production between *P3×Akaz* F1 and the first generations of backcross to *Akaz*. This strongly supports that the recombination rate is lower in asexuals compared to their closest sexual relative *Akaz*, which already has an exceptionally low recombination rate compared to other sexual species (Haag et al., 2017). Still, recombination rate is not zero. A key limiting factor in preventing the complete loss of recombination in *Ap2n* could perhaps be positive selection for rare sex or contagion, which requires residual male production and therefore nonzero recombination.

### CONTAGIOUS ASEXUALITY VERSUS RARE SEX VERSUS CYCLICAL PARTHENOGENESIS

Contagious asexuality has always been assumed to occur only via asexually produced males (in *Artemia* and other asexual species), mainly because asexual females are thought to be unable to reproduce sexually. We found, for the first time, that hybrid females with a “mixed” reproductive mode can sexually transmit asexuality. This led us to ask whether *Ap2n* females were capable of sex, although Browne and Hoopes (1990) found no evidence for cyclical parthenogenesis. To our surprise, we found that females from field‐sampled asexual lineages can rarely reproduce sexually, likely through normal meiosis, which is an entirely new result for asexual *Artemia*. It is, however, unlikely that these occurrences of rare sexual reproduction represent “cyclical parthenogenesis,” where sex occurs periodically, triggered by environmental cues (Burt, 2000; Meirmans et al., 2012) and often is linked to the production of diapause stages. Our experiment was conducted in the laboratory under controlled and constant conditions, thus dependence on environmental cues is unlikely (although we cannot exclude it). Moreover, offspring produced were live nauplii, not diapause stages (cysts). Overall, it thus seems likely that our observations represent somewhat unpredictable events of rare sexual reproduction of *Ap2n* females rather than cyclical parthenogenesis. Although the frequency of these events is unknown, these findings challenge the robustness of the evidence for purely asexual reproduction also in other taxa: Identifying occasional sex via morphologically distinct rare males is far easier than detecting rare sexual events in otherwise asexual females. Hence, it is possible that the common view that contagious asexuality occurs mainly through males may be influenced by an ascertainment bias between sexes in the study of rare sex.

### THE DIVERSIFICATION OF ASEXUAL LINEAGES

The demonstration that asexuality can be sexually transmitted also via females indicates that new asexual lineages may be created more easily than previously thought. Furthermore, contagion generates hybrids with mixed reproduction, which can in turn breed with other hybrids or backcross, thus potentially generating numerous new asexual lineages from just a single original hybridization event. Finally, rare sex may allow gene flow between different asexual lineages without the need to hybridize first with a sexual species. Even if extremely rare, this vastly expands the possibility of generating new asexual lineages as it does not require local co‐occurrence of closely related sexual species whose geographic distributions are narrow (Muñoz et al., 2010). These possibilities, combined with LOH, could explain the high diversity of asexual lineages observed in *Artemia* (Browne & Hoopes, 1990). Many asexual taxa indeed show surprisingly high genetic diversity (Parker, 1979; Browne & Hoopes, 1990; Simonsen & Holmstrup, 2008; Bengtsson, 2009). It seems likely that part of this diversity may be explained by rare occurrences of sex and recombination, although other factors may contribute (e.g., the origin of asexual lineages and mutation accumulation; Simon et al., 2003).

### THE GENETIC BASIS OF ASEXUALITY

The reproductive modes of sex‐asex hybrid females are more complex than expected. Almost all F1 females were able to reproduce asexually, which radically differs from results by Maccari et al. (2014), which suggested recessive inheritance of asexuality. Although our methodology was similar, their assumption that only sexual reproduction occurs once F1 females are paired with males was clearly rejected by our paternity analyses. In addition, some clutches in their experiment (produced in the presence of males) showed low sex ratios typical of asexual reproduction, suggesting that at least some asexual reproduction did in fact occur. Although we cannot currently explain why none of their F1 females reproduced during the 14 days of isolation period in their experiment (a substantial fraction of F1 females did so during the same period in our experiment), it is nonetheless likely that the different results and conclusions are largely explained by methodology. Our results indicate that the capacity to reproduce asexually is dominant and was homozygous in the rare males used for the crosses. Dominant asexuality is mainly found in plants (Van Dijk, 2009; Neiman et al., 2014), whereas recessive asexuality is found in several animal systems (Jaquiéry et al., 2014; Sandrock & Vorburger, 2011; Yagound et al., 2020). Together with *Daphnia* (Lynch et al., 2008), *Artemia* seem to be an exception to this pattern. Dominant asexuality also allows reversal to sexuality if LOH occurs at a heterozygous asexuality‐determining region (thus LOH is a possible explanation for the occurrence of rare sex in females as well as of the production of rare males). Surprisingly, we also found two distinct reproductive phenotypes within F1 females, which were either completely asexual or “mixed.” This could be explained by a dominant factor, heterozygous in the *Akaz* females used for the cross or by an epistatic interaction with a second locus. The two traits (the capacity to reproduce asexually and the ability to switch between sexual and asexual clutches) displayed by F1 females thus could be controlled by different loci. The occurrence of at least two loci is further suggested by the observation that the propensity to reproduce asexually in “mixed” females differs between crosses. This finding indicates that the “asexuality” phenotype may be more complex, with a history of secondary modifications, involving more than a single gene.

### SEX‐TO‐ASEX TRANSITIONS AND THE MAINTENANCE OF SEX

Over the last two decades, the classical view of regarding asexuality as largely synonymous with clonality has started to change (Gorelick, 2003; Gorelick & Carpinone, 2009; Dukić et al., 2019). Nonclonal asexual modes have been uncovered in an increasing number of taxa (Stenberg & Saura, 2009; Hiruta et al., 2010; Svendsen et al., 2015). In addition, it has become clear that even the ones that do show fully or largely clonal reproduction from a genetic point of view often do so by modified meiosis (e.g., central fusion automixis with very little or no recombination) rather than by mitosis. This suggests that recombination may have been frequent during earlier phases of their asexuality evolution (Archetti, 2010). Especially during these phases, the evolutionary consequences of asexuality may have strongly differed from those of clonality, as a different set of costs and benefits apply to nonclonal asexual modes (Stenberg & Saura, 2009; Archetti, 2010; Meirmans et al., 2012; Engelstädter, 2017). These different costs and benefits may strongly affect the fitness of new asexual lineages compared to sexual ancestors, and hence the rate at which new lineages are produced as well as their diversity. For instance, a low rate of sex in asexuals may be sufficient to confer most advantages of sexual reproduction while minimizing the cost of sex (Bengtsson, 2009; Otto, 2009; Schurko et al., 2009; Engelstädter, 2017). However, as we show, nonclonal asexuality can appear as clonality, which is too often considered as the “default” asexuality mode. This causes our conceptions of sex‐to‐asex transitions and maintenance of sex to largely rely on contrasting the costs and benefits of sex with those of clonality (Maynard Smith, 1978; Hartfield & Keightley, 2012). If generalized, this means that the twofold advantage of an asexual mitotic mutant (Maynard Smith, 1978; Hartfield & Keightley, 2012) may often simply be irrelevant (see also Meirmans et al 2012). This calls for a more realistic consideration of sex‐to‐asex transitions in theories dealing with the maintenance of sex.

## CONFLICT OF INTEREST

The authors declare no conflict of interest.

## AUTHOR CONTRIBUTIONS

TL and CH acquired funding, conceived and supervised the study. TL, CH, and LB designed the experiments. RZ and LB performed the experiments. TL provided resources. LB, MM, and TL analyzed the data. LB and MM wrote the original draft of the manuscript. TL, CH, and LB reviewed and edited the manuscript. LB and MM produced figures. TL and RZ administered the project.

## DATA ARCHIVING

Data for experiments 1 and 2 are available in Tables S9 and S10, respectively.

## Supporting information




**Figure S1**. Hybrid asexual lineages used in experiment 1 and LOH events.
**Figure S2**. Variance in rare male production among the asexual females generated by hybridization and backcross of experiment 5.
**Table S1**. Models fitted to the data of experiment 1 and ΔAICc.
**Table S2**. Reproductive mode of F1 hybrid females in each of the two crosses of experiment 2.
**Table S3**. Likelihood models fitted to the reproductive mode data of F1 hybrid females in experiment 2 and their ΔAICc
**Table S4**. Reproduction of females isolated from each candidate clutch in experiment 3.
**Table S5**. Microsatellite genotypes of potential parents (*P1* females and *Akaz* males) and male offspring from the *P1* mass‐cross in experiment 4.
**Table S6**. Sample sizes of individuals used for crosses and asexuality tests in each generation of experiment 5 and sex ratios of asexually produced offspring.
**Table S7**. Likelihood models fitted to the data on the proportion of asexually produced males in experiment 5 and their QAIC.
**Table S8**. Summary of the five experiments conducted in this study and their main results.
**Table S9**. Data for experiment 1.
**Figure S10**. Data for experiment 2.
