# Supplementary material for: Not so clonal asexuals: Unraveling the secret sex life of Artemia parthenogenetica
Source: Evol Lett. 2021 Feb 8;5(2):164–74. doi: 10.1002/evl3.216 (PMC8045904; doi:10.1002/evl3.216)
Supplement: Supplementary file 1 — Figure S1. Hybrid asexual lineages used in experiment 1 and LOH events. Figure S2. Variance in rare male production among the asexual females generated by hybridization and backcross of experiment 5. Table S1. Models fitted to the data of experiment 1 and ΔAICc. Table S2. Reproductive mode of F1 hybrid females in each of the two crosses of experiment 2. Table S3. Likelihood models fitted to the reproductive mode data of F1 hybrid females in experiment 2 and their ΔAICc Table S4. Reproduction of females isolated from each candidate clutch in experiment 3. Table S5. Microsatellite genotypes of potential parents (P1 females and Akaz males) and male offspring from the P1 mass‐cross in experiment 4. Table S6. Sample sizes of individuals used for crosses and asexuality tests in each generation of experiment 5 and sex ratios of asexually produced offspring. Table S7. Likelihood models fitted to the data on the proportion of asexually produced males in experiment 5 and their QAIC. Table S8. Summary of the five experiments conducted in this study and their main results. Table S9. Data for experiment 1. Figure S10. Data for experiment 2. [file EVL3-5-164-s001.docx]

SUPPLEMENTARY MATERIALS

1. **Origin of laboratory populations and culturing conditions**

The *P1* population was established from a single female sampled in the Aigues-Mortes salterns, France by Nougué et al. (2015) (referred to as PAM7 in that publication). A second population, *P3*, also sampled from Aigues-Mortes but from a different location and not isofemale was used in experiment 5. The *P2* population originated from Lake Urmia, Iran, with cysts obtained from F. Amat (Instituto de Acuicultura de Torre de la Sal, Spain). Note that this is an *Ap2n* population, different from the bisexual species *A. urmiana*. The mitochondrial haplotypes of P1 (unpublished data) and P2 (Maccari et al., 2013) are closest to *Akaz*. The *Akaz* population originated from Kazakhstan, and cysts were obtained from the *Artemia* Reference Center (Ghent University, Belgium, code of the cyst sample: ARC1039). The *P2* and *P3* populations were maintained in the laboratory for <1 year (<12 generations), and the *P1* and *Akaz* populations for >5 years (>60 generations) before the beginning of the experiments. Population cultures of >100 individuals were maintained at 21 ± 1 °C in 5 L aquaria in a solution of brine diluted with deionized water to a salinity of 80 to 90 g/L. They were fed *ad libitum* with freeze-dried microalgae *Tetraselmis chuii* diluted in deionized water. *Ap2n* population cultures were regularly scanned for males (recognizable by their claspers). These males were crossed with virgin *Akaz* females. Individual juveniles, adults, pairs and their offspring were kept in plastic jars in 100 mL of brine solution (as above). They were fed three times a week with 1-2 mL of 1 g/L freeze-dried *Tetraselmis chuii* diluted in deionized water.

1. **Experiment 1: LOH in sex-asex hybrids**

We produced three crosses between *P1* rare males and *Akaz* females (two using the same rare male, Fig. S1), and two crosses between *P2* rare males and *Akaz* females (Fig. S1). From each cross, one F1 female was isolated at juvenile stage and kept individually until she produced offspring, which were in turn isolated as well. Lineages, which were sometimes split into sub-lineages, were propagated in this way for one to few generations. One lineage from each cross was selected for the experiment, which lasted up to 13 asexual generations (Fig. S1).

DNA extractions for microsatellite analysis was carried out using the digestive tracts of dissected females, which contain sufficient DNA for microsatellite analysis. Extraction methods and protocols for microsatellite analyses followed those described by Nougué et al. (2015), which were based on two previous studies (Muñoz et al., 2009; Nougué et al., 2015). DNA was extracted in 15 µL HotShot extraction buffer (Sigma), in a thermocycler at 95 °C for 10 min and 20 °C for 10 min. The extraction product was then diluted with 25 µL sterile water. We added 1 µL of DNA extract to 9 µL of a PCR mix containing 5 µL of Multiplex buffer (Qiagen), 1 µL of forward and 1 µL of reverse primers [2 µM], and 2 µL of sterile water. PCR amplification was conducted in a thermocycler with an initial denaturation step at 95 °C for 15 min, 30 cycles consisting of 30 s at 94 °C, 90 s at 60 °C and 60 s at 72 °C, and finally a supplementary extension step of 30 min at 60 °C.

We added 3 µL of diluted PCR product (20 to 200X) to 15 mL of HI-DI TM formamide (Applied Biosystems) and 0.2 mL of GeneScan-500 LIZ size standard. Samples were analysed on an ABI 3500XL 24 capillary sequencer DNA Analyzer (Applied Biosystems) at the LabEx CeMEB sequencing platform (Montpellier, France). Fragment analysis and scoring were carried out using GeneMapper v. 3.7 (Soft Genetics, State College, PA, USA). The DNA fragment sizes measured from the peaks were converted into discrete alleles by comparison with reference lists of allele sizes.

Only six loci were informative in our study: L1: Appm20, L2: Apcpm1, L3: Aupm16, L4: Aupm21 L5: AP02, and L6: AP01, with locus numbers corresponding to Figure 1 and Figure S1 and locus names to those given in Nougué et al. (2015). Note that locus 4 was only initially heterozygous in one F1 female and was lost at the third generation, thus it was not included in the statistical analyses. We also observed three LOH events in *P2xAkaz* lineages for a seventh locus, AP03, which was heterozygous with a null allele. This locus was non-informative as homozygosity was only detectable for the null allele. In some cases, the loci were analyzed in simplex reactions rather than in the multiplex reactions described in Nougué et al. (2015).

To test whether LOH rate depends on the population of origin of the rare male (“*pop*”), differs among loci (“*loc*”), or changes across subsequent asexual generations (“*gen*”), we considered several likelihood models, written and then fitted using Mathematica V.9.0 (Wolfram Research, 2012; Table S1). The likelihood of each model was written by supposing that the loss or retention of heterozygosity from one generation to the next resulted from a Bernoulli draw. In the first model, the rate of LOH was assumed to be constant across generations and loci. In the second model, it was allowed to vary with the number of asexual generations in a logistic way. The third and fourth models allowed for different LOH rates depending on the origin of the rare male (*P1* or *P2*) and/or depending on the locus, and subsequent models included combinations of these effects as well as interactions. We compared models using Akaike’s information criterion with a correction for small sample sizes (AICc). Models were checked for, but did not show overdispersion (ĉ = 0.39).

1. **Experiment 2: Reproductive mode of F1 hybrid females**

We crossed each of two rare males, one from *P1* and one from *P2*, with virgin *Akaz* females. All F1 hybrids were individually isolated at juvenile stage to assure that they remained unmated. They were sexed according to morphological characters (claspers for males, ovisac for females). Adult females were kept isolated to check for asexual reproduction. The generation time in *Artemia* is about a month. F1 Females from the *P2*x*Akaz* cross were kept isolated (period 1) for 14 days, while females from the *P1*x*Akaz* cross were isolated for 30 days to increase the possibility of asexual reproduction. After the isolation period, they were each paired with an *Akaz* male (period 2), and couples were kept together for up to 70 days (dead males were replaced by new ones). Offspring produced during period 2 were subjected to paternity testing to determine whether they were produced sexually or asexually.

In sexual and asexual *Artemia*, offspring are produced in clutches of either liveborn nauplii or cysts (Browne, 1992), which in this experiment were treated similarly (i.e., both were considered successful reproduction events). Pairs were checked three times a week for clutches, and each individual clutch produced was collected. Live nauplii were grown to juvenile stage and then preserved in 96 % ethanol at -20 °C. Cysts were dried and kept at -3 °C. Once a pair had produced three (*P2*x*Akaz* cross) or six (*P1*x*Akaz* cross) clutches, or if the female displayed signs of poor viability, adults were also sampled and preserved in 96 % ethanol at -20 °C. A higher clutch number threshold was applied to the *P1*x*Akaz* cross, which was performed after *P2xAkaz*, to increase the chance of observing both reproductive modes (sexual and asexual) during pairing in this second cross. DNA extraction for microsatellite paternity tests were carried out using whole juveniles or cysts for the offspring and half bodies or digestive tracks for the adults.

We conducted microsatellite paternity tests on a total of 63 pairs and 167 clutches (Table S2). We used the same protocols as described in section 1. For extractions, we used whole samples when they were small (such as juveniles, digestive tracts and cysts), and half of large samples (adult individuals). Before extraction, cysts were rehydrated during one hour in 100 µL sterile water, then crushed directly in the extraction buffer. We used the multiplex reaction described in Muñoz et al. (2009), which includes the loci with highest divergence between *Akaz* and *Ap2n* and hence most likely to be informative regarding paternity. For a subset of 21 clutches (with three to eight offspring each), all offspring were analyzed, and the results indicated that, within a clutch, all offspring were produced by the same reproductive mode (sexual or asexual). We assumed this to be the case also for the remaining clutches and therefore, carried out paternity tests on only two offspring per clutch for each of the remaining clutches (in every case, both were indeed produced by the same reproductive mode).

Paternity testing accounted for the possible presence of null alleles. For instance, a locus with a homozygous profile for different alleles in the two parents and a homozygous profile for the maternal allele in a given offspring were counted as non-informative, as it is possible that the father was, in fact, heterozygous for a null allele and passed that null allele via sexual reproduction to its offspring. Due to these uncertainties, we could reliably infer the mode of reproduction for only 81 % of all clutches that were produced. But for all except four of the 63 females that were paired with a male, the mode of reproduction could be inferred for at least one of their clutches.

To analyze the proportions of the different modes of reproduction in F1 females, we concentrated only on the period when females were paired with a male because only during this period both reproductive modes were possible. We conducted a likelihood analysis, which accounted for sampling effects. We considered three categories of models (Table S3), written and then fitted using Mathematica V.9.0 (Wolfram Research, 2012). Models of category A assumed that all F1 females were 'mixed', and that the proportion of asexually produced clutches was a constant. The probability of observing a given number of asexual clutches then followed a binomial distribution. Because the data shows a large proportion of asexual clutches (Table S2), models of categories B and C considered two types of F1 females, asexual ones and mixed ones. We introduced a parameter to measure the proportion of these two types of females. In models of category B, the proportion of asexually produced clutches in mixed females was constant, but in models of category C, we assumed that mixed females were heterogeneous and that the proportion of asexually produced clutches followed a beta distribution among females. In all cases, we further considered a possible effect of the cross (*P1* or *P2*) on the probability to reproduce asexually. In model C.3, we considered an effect of the cross on the mean of the beta distribution but not on the variance. In models of categories B and C, we also considered an effect of the cross on the probability to be in the 'mixed' category. We then compared models using AICc. The best model, model B.2 was slightly underdispersed (ĉ = 0.88). An additional model suggested that there was no evidence for the existence of F1 females with 100% sexual reproduction: the model was identical to model B.2, except that it included three categories of females (mixed, 100% asexual and 100% sexual). To avoid bias, both model B.2 and the three-categories model were fitted to a dataset omitting the six mixed females that reproduced asexually during period 1 but only reproduced sexually during period 2 (Table S2). Again, model B.2 performed better (ΔAICc=2.1).

1. **Experiment 3: Contagious asexuality *via* hybrid F1 females**

In order to determine whether asexuality could be sexually transmitted by mixed hybrid F1 females, we kept large clutches of nauplii produced by F1 females from the *P1*x*Akaz* cross in experiment 2 after these F1 females were paired with *Akaz* males to test for sexual reproduction. As we did not yet know which clutches were produced sexually, we used the sex ratio of these clutches (as morphologically assessed once the offspring reached pre-adulthood) as a first indication. Indeed, sexually produced clutches tend to have a much higher proportion of male offspring than asexually produced ones. Twelve clutches with one or more males were selected as candidates. After paternity testing, five of these were found to be indeed sexually produced. One to six virgin females of each of these clutches were isolated, and checked weekly for offspring production for four weeks. Two females originating from the same clutch produced cysts in isolation, demonstrating that they were able to reproduce asexually (Table S4). The fact that contagious asexuality was observed in only one out of five clutches tested, is possibly explained by the nature of the cross, a backcross to the sexual species, which may, in some cases, lead to a loss of asexuality-conferring genes. This experiment was not conducted on the *P2xAkaz* cross, which was performed before the *P1xAkaz* cross, because at that time we did not know that some F1 females had a mixed reproductive mode.

1. **Experiment 4: Rare sex in *Ap2n* females**

We grew large *P1* and *P2* isofemale lines (i.e*.*, populations of asexual descendants of a single female), and placed all healthy females in 5L aquaria (one aquarium per line). We added young *Akaz* males so that the ratio of *Akaz* male to *Ap2n* female was approximately equal for each population. The first aquarium contained 115 females from the *P1* isofemale line with 57 *Akaz* males, while the second contained 52 females from the *P2* isofemale population with 25 *Akaz* males. The experiment lasted for four weeks, and each week we moved all parents to a new aquarium to allow their offspring to grow with reduced competition. At the end of the experiment, all parents were preserved in 96 % ethanol at -20 °C. The offspring produced during the experiment were morphologically sexed once they reached pre-adulthood. In total, sexing revealed 1820 female and 8 male offspring from the *P1* isofemale population and 1057 female and 4 male offspring from the *P2* isofemale population. After sexing, offspring were also preserved in 96 % ethanol at -20 °C.

All male offspring as well as eight female offspring, two female parents and five male parents from each population were used for microsatellite analyses, using the same protocols as described in section 1 and the same multiplex reaction as in section 2. We used half bodies for DNA extraction. We determined whether the offspring were produced sexually or asexually using the same criteria as described in section 2.

1. **Experiment 5. Estimating recombination rate in automicts**

From an initial cross between a *P3* rare male and an *Akaz* female, we obtained a F1 and four backcross generations on *Akaz*, using two to three *P3xAkaz* males per each generation, crossed again to *Akaz* females (Table S6). These males were either sexually produced during the backcross itself or asexually by females from a given backcross generation. We maintained asexuality throughout these generations, by using males that were most likely to carry asexuality genes, as indicated by the fact that sisters of these males were able to reproduce asexually. This was assessed by isolating females before they reached adulthood and by keeping them isolated for 30 to 45 days once adult to check for asexual reproduction. We confirmed that the males actually transmitted asexuality-conferring genes by verifying that some of their daughters were also able to reproduce asexually. Sub-lineages in which asexuality was not transmitted (or where results were inconclusive) were discarded. Note that maintaining asexuality throughout these generations was only possible by these selection procedures. Indeed, in every generation, a majority of sub-lineages were discarded, though to prove that discarded females were sexuals would have required pairing them with a genetically distinct male and carrying out paternity tests, which was not done. In each generation, all offspring produced during the asexuality tests were sexed using morphological characters once they reached pre-adulthood. We refer to the proportion of males in these data as the proportion of asexually produced males ($\alpha$) and used it as a proxy for recombination rate in each generation.

To analyze the sex-ratio data, we fitted 21 likelihood models to the data and assessed their relative fit (Table S7). We used two categories of models: In the first category, “monogenic models” (category A in Table S7), we assumed that recombination rate differences between *Ap2n* and *Akaz* are governed by a single major locus with two alleles. In the second category, “polygenic models” (category B in Table S7), we assumed that these differences are under a polygenic control. In monogenic models, we assumed that F1 are heterozygous (+/-), with the allele for higher recombination (allele +) coming from *Akaz* and the allele for lower recombination (allele -) from *Ap2n*. Hence, in backcross females, there are only two possible recombination genotypes (+/- or +/+), and thus two possible sex-ratio phenotypes in their progeny, *p_1_* for (+/-) females and *p_2_* for (+/+) females. In the model we thus considered that offspring sexes were sampled from either of two binomial distributions *B*(*Φn*, *p_1_*) or *B*((1-*Φ*)*n*, *p_2_*), with *n* being the total number of offspring, *Φ* being the probability to sample from one or the other distribution (i.e., whether an offspring is +/- or +/+), *p_1_* being the probability to produce a male for (+/-) females and *p_2_* for (+/+) females, re-parameterized such that *p_2_* = *p_1_*+*f*(1-*p_1_*), where parameter *f* is defined within a constant range 0 < *f* < 1. Different variants of this model considered that parameters were either constant or varied across generations, in a linear or stepwise manner (the latter corresponding to an initial increase in sex ratio, from generation F1 to backcross 1, followed by a plateau). The polygenic models assumed that offspring sexes were also binomial samples, but the proportion of this binomial was assumed to vary among females at each generation. Specifically, this proportion was assumed to follow a Beta distribution (as in BetaBinomial models often used to model extra-binomial variation for overdispersed data; Harrison, 2015). In this way, different females could exhibit different offspring sex-ratio (i.e., different rates of rare male production) in a continuous way, as if the trait was polygenic and continuously varying among females at a given generation. In these models, the Beta distribution was parameterized with its mean (*µ,* the average offspring sex-ratio among females) and variance (*V*, the heterogeneity among females for this sex-ratio). Different constraints on parameter values were investigated to determine if the mean (*µ*) and variance (*V*) of sex-ratio changed across backcross generations following a linear, step, quadratic and/or cubic variation. For each model, the likelihood of the data was written and then fitted using Mathematica V.9.0 (Wolfram Research, 2012). Model comparison was done using quasi Akaike information criterion (QAIC). The best model (B.6) explained 44 % of the total deviance and was moderately overdispersed (ĉ = 1.7).

Given the proportion of males observed among asexual offspring of a female ($\alpha$), it is possible to compute the proportion of heterozygosity loss (*k*) that would explain the observed rate of male production

| $\alpha=\frac{(k/2)}{(1-k)+(k/2)}$ | (1) |
| --- | --- |

The numerator represents the proportion of males in the zygotes. This is given by the rate of the loss of heterozygosity divided by two, as only half of the homozygous individuals are ZZ and thus, males. This proportion is divided by the total number of surviving individuals, which is given by one minus the loss of heterozygosity, representing the proportion of heterogametic female individuals (ZW, assuming the WW genotype generated by recombination is lethal), plus the males (ZZ). This ratio provides the proportion of male offspring produced given a level *k* of heterozygosity loss. Under automixis, it is possible to compute the expected loss of heterozygosity for a given genetic distance from the centromere (Svendsen et al., 2015). Under central fusion automixis, this LOH rate is zero at the centromere and increases up to 33 % for a large genetic distance. When sexual genome was introgressed in asexual *Artemia*, $\alpha$ reached the value of 0.27, which gives an estimate of 42% loss of heterozygosity. This LOH rate exceeds the maximal LOH rate expected under central fusion automixis (i.e., for a locus at a large genetic distance from the centromere), although not significantly (Figure 3).

SUPPLEMENTARY REFERENCES

Harrison, X. A. (2015). A comparison of observation-level randomeffect and Beta-Binomial models for modelling overdispersion in Binomial data in ecology & evolution. *PeerJ*, *2015*(7). https://doi.org/10.7717/peerj.1114

Maccari, M., Amat, F., & Gómez, A. (2013). Origin and Genetic Diversity of Diploid Parthenogenetic Artemia in Eurasia. *PLoS ONE*, *8*(12). https://doi.org/10.1371/Citation

Muñoz, J., Green, A. J., Figuerola, J., Amat, F., & Rico, C. (2009). Characterization of polymorphic microsatellite markers in the brine shrimp Artemia (Branchiopoda, Anostraca). *Molecular Ecology Resources*, *9*(2), 547–550. https://doi.org/10.1111/j.1755-0998.2008.02360.x

Nougué, O., Flaven, E., Jabbour-Zahab, R., Rode, N. O., Dubois, M. P., & Lenormand, T. (2015). Characterization of nine new polymorphic microsatellite markers in Artemia parthenogenetica. *Biochemical Systematics and Ecology*, *58*, 59–63.

Nougué, O., Rode, N. O., Jabbour-zahab, R., Ségard, A., Chevin, L. M., Haag, C. R., & Lenormand, T. (2015). Automixis in Artemia: Solving a century-old controversy. *Journal of Evolutionary Biology*, *28*(12), 2337–2348. https://doi.org/10.1111/jeb.12757

Svendsen, N., Reisser, C. M. O., Dukić, M., Thuillier, V., Ségard, A., Liautard-Haag, C., Fasel, D., Hürlimann, E., Lenormand, T., Galimov, Y., & Haag, C. R. (2015). Uncovering cryptic asexuality in daphnia magna by RAD sequencing. *Genetics*, *201*(3), 1143–1155. https://doi.org/10.1534/genetics.115.179879

Wolfram Research, I. (2012). *Mathematica* (9.0). Wolfram Research, Inc.

SUPPLEMENTARY TABLES AND FIGURES


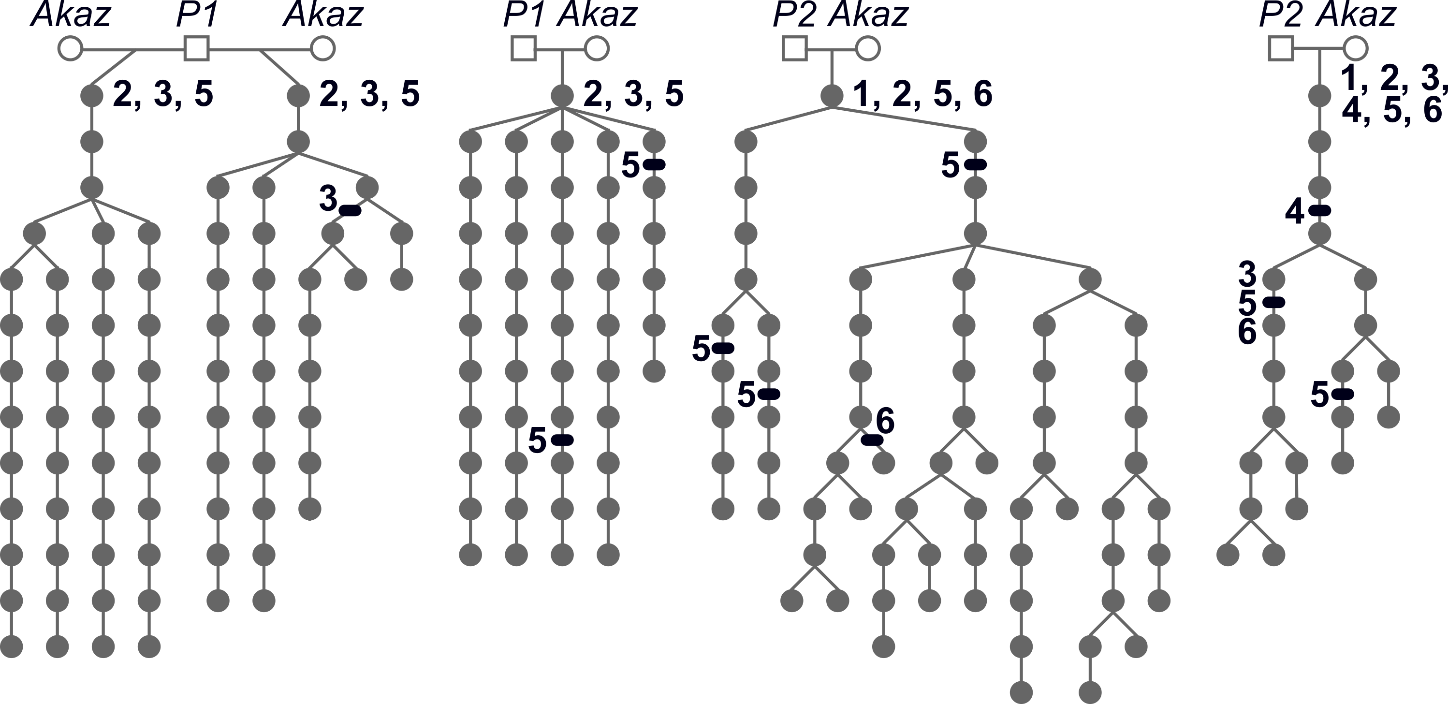


**Figure S1. Hybrid asexual lineages used in experiment 1 and LOH events.** The figure shows the genealogy of all lineages and sub-lineages starting from *P1* or *P2* rare males (white squares) crossed with *Akaz* females (white circles). Each grey dot corresponds to an F1 female and asexually produced further-generation descendant of the F1 females. Some lineages had not reached 13 generations at the time of the analysis or they were stopped earlier. Informative loci (1 to 6) that were initially heterozygous are indicated next to each F1 female. The seventh locus is not represented due to its ambiguity. LOH events are represented by black bars with the corresponding locus number next to them.


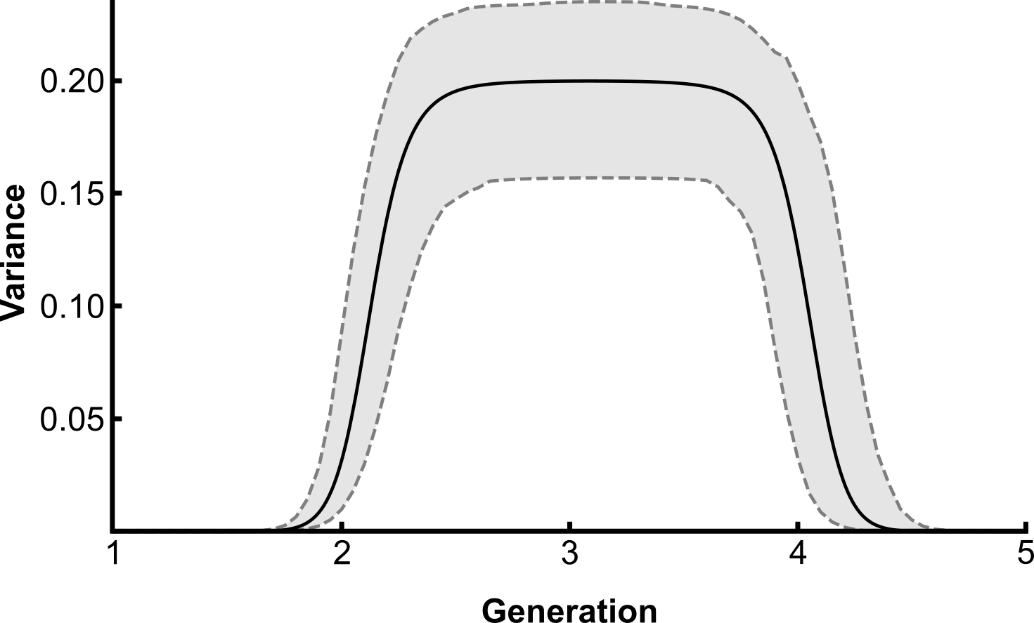


**Figure S2. Variance in rare male production among the asexual females generated by hybridization and backcross of experiment 5.** The thick black line represents the variance as estimated by the best model and the grey dotted lines represent the support limits.

| Name | Model | ΔAICc |
| --- | --- | --- |
| 1 | *p_i_* ~ constant | 19.2 |
| 2 | *p_i_* ~ *gen* | 19.5 |
| 3 | *p_i_* ~ *pop* | 17.7 |
| 4 | *p_i_* ~ *loc* | 11.0 |
| 5 | ***p_i_* ~ *loc* + *pop*** | **0** |
| 6 | *p_i_* ~ *loc* * *pop* | 9.5 |
| 7 | *p_i_* ~ *loc* + *gen* + *pop* | 2.2 |
| 8 | *p_i_* ~ *loc* * *gen* + *pop* | 11.3 |
| 9 | *p_i_* ~ *loc* + *gen* * *pop* | 3.6 |

**Table S1.** **Models fitted to the data of experiment 1 and ΔAICc.** *p_i_* represents the LOH rate, “*gen*” is the number of generations since the F1, “*pop*” is the population of origin of the rare male (*P1* or *P2*), and “*loc*” is the locus. The best model, model 5, is highlighted in bold. ΔAICc are given relative to this model.

|  | | Cross | |
| --- | --- | --- | --- |
|  | | *P1xAkaz* | *P2xAkaz* |
| Number of F1 offspring | Males | 61 | 84 |
|  | Females | 51 | 85 |
| Isolation period | Reproduction | 39 | 38 |
|  | No reproduction | 5 | 47 |
| Pairing with *Akaz* males | Asexual reproduction | 19 | 20 |
|  | Sexual reproduction | 2 | 9 |
|  | Mixed reproduction | 5 | 4 |
|  | NA | 2 | 2 |

**Table S2. Reproductive mode of** **F1 hybrid females in each of the two crosses of experiment 2.** The first section (Number of F1 offspring) indicates the number of male and female F1 individuals produced by each cross. The second section (Isolation period) refers to the number of females that reproduced or not during the isolation period (30 days for the *P1*x*Akaz* cross, 14 days for the *P2*x*Akaz* cross), conditional on survival for the entire isolation period. The third section (Pairing with *Akaz* males) indicates the number of F1 females that produced clutches only by asexual reproduction, only by sexual reproduction or that produced subsequent clutches by both modes of reproduction (‘mixed’) when paired with *Akaz* males. NA refers to the number of F1 females that produced clutches when paired with *Akaz* males, but for which the mode of reproduction could not reliably be assessed. The females that did not reproduce in the presence of a male are not included in the third section. Of the females that only produced sexual clutches wile paired, both females from the *P1xAkaz* cross and four of the nine females from the *P2xAkaz* cross did reproduce asexually while isolated. These females thus had a mixed reproductive mode.

| Model | Proportion of mixed F1 females | | | Proportion of sexually produced clutches in mixed F1 females | | | | | |  |
| --- | --- | --- | --- | --- | --- | --- | --- | --- | --- | --- |
|  |  |  |  | Constant | | Beta | | | |  |
|  | 1 | *q* | *q*~cross | *p* | *p*~cross | *ɑ* | *β* | *ɑ*~cross | *β*~cross | ΔAICc |
| A.1 | X |  | | X |  |  | | | | 19.8 |
| A.2 | X |  |  |  | X |  |  |  |  | 12.0 |
| B.1 |  | X |  | X |  |  |  |  |  | 7.3 |
| **B.2** |  | ***q* = 0.44** |  |  | ***P1*: *p* = 0.29; *P2*: *p* = 0.79** |  |  |  |  | **0** |
| B.3 |  |  | X | X |  |  |  |  |  | 6.5 |
| B.4 |  |  | X |  | X |  |  |  |  | 2.1 |
| C.1 |  | X |  |  | |  |  | X | X | 3.9 |
| C.2 |  |  | X |  |  | X | X |  |  | 3.6 |
| **C.3** |  | ***q* = 0.52** |  |  |  |  |  | ***P1* Mean *p* = 0.25 ;**  ***P2* Mean *p* = 0.70 ;**  **Variance in *p* = 0.05** | | **1.1** |

**Table S3. Likelihood models fitted to the reproductive mode data of F1 hybrid females in experiment 2 and their** **ΔAICc** Models of category A allow for mixed females only, while models from categories B and C allow for asexual and mixed females, with *q* being the proportion of mixed females. In mixed females, *p* is the proportion of sexually produced clutches, and *ɑ* and *β* are the parameters of the beta distribution modelling the distribution of *p*, when applicable. The effect of the cross (*P1xAkaz* or *P2xAkaz*) is represented by *~cross*. The best model (in bold) is B.2. The parameter estimates of this model are indicated, with *P1 and P2* being F1 females from the *P1* and *P2* crosses, respectively. Model C.3 (also in bold) fits the data only slightly worse, with ΔAICc < 2 compared to B.2. Its interpretation is however very close, simply adding some biological heterogeneity in the rate of asexuality among mixed females (their fitted average is very close to the point estimate in model B.2). For each model, the cross X indicates parameters that are estimated in each model. Empty cells indicate parameters that were not estimated and greyed-out zones indicate that the parameters are not relevant for the model.

| Candidate clutch number | Clutch produced by | N females isolated | N female that reproduced |
| --- | --- | --- | --- |
| 1 | Asexual reproduction | 1 | 1 |
| 2 | Asexual reproduction | 1 | 1 |
| 3 | Asexual reproduction | 2 | 2 |
| 4 | Asexual reproduction | 1 | 1 |
| 5 | Asexual reproduction | 1 | 1 |
| 6 | Asexual reproduction | 6 | 6 |
| 7 | Asexual reproduction | 3 | 0 |
| 8 | Sexual reproduction | 3 | 0 |
| 9 | Sexual reproduction | 1 | 0 |
| 10 | Sexual reproduction | 3 | 0 |
| **11** | **Sexual reproduction** | **2** | **2** |
| 12 | Sexual reproduction | 1 | 0 |

**Table S4.** **Reproduction of females isolated from each candidate clutch in experiment 3.** Clutches later identified (by paternity analysis) as sexual are shown in grey. Females from clutch 11 (in bold) were able to reproduce asexually (production of cysts).

| Microsatellite marker | AP01 | | AP02 | | AP03 | | Offspring produced by |
| --- | --- | --- | --- | --- | --- | --- | --- |
| *P1* female 1 (potential mother) | 165 | 0 | 221 | 224 | 0 | 0 |  |
| *P1* female 2 (potential mother) | 165 | 0 | 221 | 224 | 0 | 0 |  |
| *Akaz* male 1 (potential father) | 185 | 185 | 221 | 221 | 202 | 214 |  |
| *Akaz* male 2 (potential father) | 187 | 187 | 221 | 236 | 214 | 214 |  |
| *Akaz* male 3 (potential father) | 187 | 187 | 221 | 236 | 214 | 214 |  |
| *Akaz* male 4 (potential father) | 187 | 187 | 221 | 221 | 214 | 214 |  |
| *Akaz* male 5 (potential father) | 187 | 187 | 221 | 236 | 214 | 214 |  |
| Male offspring 1 | 165 | 0 | 221 | 224 | 0 | 0 | Asexual reproduction |
| **Male offspring 2** | **165** | **185** | **221** | **221** | **202** | **0** | **Sexual reproduction** |
| Male offspring 3 | 165 | 0 | 221 | 224 | 0 | 0 | Asexual reproduction |
| **Male offspring 4** | **185** | **0** | **221** | **224** | **202** | **0** | **Sexual reproduction** |
| **Male offspring 5** | **185** | **0** | **221** | **224** | **214** | **0** | **Sexual** **reproduction** |
| **Male offspring 6** | **165** | **185** | **221** | **224** | **202** | **0** | **Sexual** **reproduction** |
| Male offspring 7 | 165 | 0 | 221 | 224 | 0 | 0 | Asexual reproduction |
| Male offspring 8 | 165 | 0 | 221 | 224 | 0 | 0 | Asexual reproduction |
| Female offspring 1 | 165 | 0 | 221 | 224 | 0 | 0 | Asexual reproduction |
| Female offspring 2 | 165 | 0 | 221 | 224 | 0 | 0 | Asexual reproduction |
| Female offspring 3 | 165 | 0 | 221 | 224 | 0 | 0 | Asexual reproduction |
| Female offspring 4 | 165 | 0 | 221 | 224 | 0 | 0 | Asexual reproduction |
| Female offspring 5 | 165 | 0 | 221 | 224 | 0 | 0 | Asexual reproduction |
| Female offspring 6 | 165 | 0 | 221 | 224 | 0 | 0 | Asexual reproduction |
| Female offspring 7 | 165 | 0 | 221 | 224 | 0 | 0 | Asexual reproduction |
| Female offspring 8 | 165 | 0 | 221 | 224 | 0 | 0 | Asexual reproduction |

**Table S5. Microsatellite genotypes of potential parents (*P1* females and *Akaz* males) and male offspring from the *P1* mass-cross in experiment 4.** The reproductive mode by which each offspring was produced, according to paternity testing, is indicated in the last column. Males produced sexually are shown in bold. In three of the four sexually produced males, one of the maternal alleles was absent (allele 224 was missing in male offspring 2 at the AP02 locus and allele 165 was missing in male offspring 4 and 5 at the AP01 locus), suggesting their mother produced haploid ovules. The genotype of the fourth male (male offspring 6) was inconclusive regarding ploidy of the ovule, due to null-alleles and shared alleles between males and females.

| Generation | N fathers | N females tested | N asexually produced female offspring | N asexually produced male offspring | Sex ratio |
| --- | --- | --- | --- | --- | --- |
| F1 | 1 | 5 | 46 | 3 | 0.06 |
| BC1 | 3 | 12 | 51 | 23 | 0.31 |
| BC2 | 2 | 3 | 23 | 0 | 0.00 |
| BC3 | 2 | 6 | 12 | 5 | 0.29 |
| BC4 | 3 | 4 | 9 | 4 | 0.3 |

**Table S6. Sample sizes of individuals used for crosses and asexuality tests in each generation of experiment 5 and sex ratios of asexually produced offspring.** *N* fathers is the number of males of the previous generation used to produce the offspring of a given generation. BC stands for backcross.

| Name | Parameters | *df* | ΔQAIC |
| --- | --- | --- | --- |
| A.1 | *p_1_*[constant], *f*[constant], *Φ*[constant] | 3 | 7.8 |
| A.2 | *p_1_*[constant], *f*[constant], *Φ*[time] | 4 | 8.4 |
| A.3 | *p_1_*[time], *f*[constant], *Φ*[time] | 5 | 10.1 |
| A.4 | *p_1_*[constant], *f*[constant], *Φ*[step] | 4 | 6.3 |
| A.5 | *p_1_*[step], *f*[constant], *Φ*[step] | 5 | 8.3 |
| A.6 | *p_1_*[gen], *f*[constant], *Φ*[step] | 8 | 7.3 |
| A.7 | *p_1_*[time], *f*[time], *Φ*[time] | 6 | 12.0 |
| A.8 | *p_1_*[constant], *f*[constant], *Φ*[gen] | 7 | 7.9 |
| A.9 | *p_1_*[gen], *f*[gen], *Φ*[gen] | 15 | 15.8 |
| B.1 | *µ* [constant], and *V*[constant] | 2 | 6.2 |
| B.2 | *µ* [time], and *V*[time] | 4 | 8.8 |
| B.3 | *µ* [step], and *V*[step] | 4 | 5.8 |
| B.4 | *µ* [step], and *V*[constant] | 3 | 6.6 |
| B.5 | *µ* [step], and *V*[time] | 4 | 7.2 |
| **B.6** | ***µ* [step], and *V*[time^2^]** | **5** | **0** |
| B.7 | *µ* [time], and *V*[time^2^] | 5 | 4.0 |
| B.8 | *µ* [constant], and *V*[time^2^] | 4 | 7.4 |
| B.9 | *µ* [step], and *V*[gen] | 7 | 4.0 |
| B.10 | *µ* [time^2^], and *V*[time^2^] | 6 | 2.9 |
| **B.11** | ***µ* [time^3^], and *V*[time^2^]** | **7** | **1.8** |
| B.12 | *µ* [gen], and *V*[time^2^] | 8 | 3.6 |

**Table S7. Likelihood models fitted to the data on the proportion of asexually produced males in experiment 5 and their QAIC.** Models of category A are the monogenic models. In these models, we suppose two categories of individuals with different rates of rare male production. these two categories are in proportion *Φ* and 1- *Φ* . The rates of rare male production in each categories are fitted using *p_1_* and *f* parameters (sup. mat. 6). [constant] indicates that the parameter is assumed to be constant across generations; [time] indicates that the parameter is fitted with an intercept and a linear effect of the number of generation; [step] indicates that the parameter is assumed to take two values, one for generation 1 and another for all subsequent generations; [gen] indicates that the parameter is fitted at different values for each generation. Models of category B are the polygenic models. Different constraints on parameter values were investigated in each case to determine if the mean (*µ*) and variance (*V*) of the sex-ratio changed across generations. The codes are the same than for the monogenic models, with, in addition, [time^2^] and [time^3^] referring to a quadratic and cubic effect of the number of generations on the corresponding parameter, respectively. Model B.6 (in bold) is the best model. Model B.11 (also in bold) fits the data only slightly worse, with ΔQAIC < 2 with respect to B.6. Its interpretation is however similar, with a cubic variation of *µ* with a qualitatively similar shape as in model B.6.

| Exp. no. | Exp. name | Principle | Main results |
| --- | --- | --- | --- |
| **1** | LOH in sex-asex hybrids | Estimate LOH at microsatellite markers in asexually reproducing sex-asex F1 hybrids. | Recombination occurs.  Its rate differs depending on markers and origin of the rare male. |
| **2** | Reproductive mode of F1 hybrid females | Determination of the reproductive mode of female sex-asex F1 hybrids using isolation tests and paternity testing of progeny from paired females. | Most if not all F1 females can reproduce asexually. Some F1 females can be mixed (i.e*.*, reproduce both sexually and asexually). |
| **3** | Contagious asexuality *via* hybrid F1 females | Test of asexual reproduction in sexually produced BC1 offspring, produced by mating between F1 hybrid females with mixed reproduction mode and *Akaz* males. | Mixed F1 females can transmit asexuality to their sexually produced daughters. |
| **4** | Rare sex in *Ap2n* females | Mass-cross between *Ap2n* females and *Akaz* sexual males; paternity testing on male offspring. | *Ap2n* females can rarely undergo sexual reproduction, likely through normal meiosis. |
| **5** | Estimating recombination rate in automicts | Production of several generations of sex-asex hybrids and backcrosses to *Akaz*, while maintaining asexuality genes; use the rate of rare male production at each generation as a proxy for recombination rate. | Introgression of the *Akaz* genome into *Ap2n* increases the recombination rate. Differences likely caused by selection against recombination in *Ap2n.* |

**Table S8.** Summary of the five experiments conducted in this study and their main results.

| Marker | Marker name | F1 genotype | Asex pop | Cross | LOH | LOH genotype | True generations | Independent generations |
| --- | --- | --- | --- | --- | --- | --- | --- | --- |
| L1 | Appm20 | 110/112 | *P2* | *P2-1 x Akaz* | no | . | 9 | 9 |
| L1 | Appm20 | 110/112 | *P2* | *P2-1 x Akaz* | no | . | 9 | 5 |
| L1 | Appm20 | 110/112 | *P2* | *P2-1 x Akaz* | no | . | 11 | 11 |
| L1 | Appm20 | 110/112 | *P2* | *P2-1 x Akaz* | no | . | 11 | 1 |
| L1 | Appm20 | 110/112 | *P2* | *P2-1 x Akaz* | no | . | 9 | 1 |
| L1 | Appm20 | 110/112 | *P2* | *P2-1 x Akaz* | no | . | 8 | 1 |
| L1 | Appm20 | 110/112 | *P2* | *P2-1 x Akaz* | no | . | 12 | 9 |
| L1 | Appm20 | 110/112 | *P2* | *P2-1 x Akaz* | no | . | 11 | 2 |
| L1 | Appm20 | 110/112 | *P2* | *P2-1 x Akaz* | no | . | 11 | 3 |
| L1 | Appm20 | 110/112 | *P2* | *P2-1 x Akaz* | no | . | 8 | 1 |
| L1 | Appm20 | 110/112 | *P2* | *P2-1 x Akaz* | no | . | 13 | 10 |
| L1 | Appm20 | 110/112 | *P2* | *P2-1 x Akaz* | no | . | 9 | 1 |
| L1 | Appm20 | 110/112 | *P2* | *P2-1 x Akaz* | NA | NA | 13 | 9 |
| L1 | Appm20 | 110/112 | *P2* | *P2-1 x Akaz* | NA | NA | 12 | 1 |
| L1 | Appm20 | 110/112 | *P2* | *P2-1 x Akaz* | NA | NA | 11 | 3 |
| L1 | Appm20 | 110/112 | *P2* | *P2-2 x Akaz* | no | . | 10 | 10 |
| L1 | Appm20 | 110/112 | *P2* | *P2-2 x Akaz* | no | . | 10 | 1 |
| L1 | Appm20 | 110/112 | *P2* | *P2-2 x Akaz* | no | . | 9 | 2 |
| L1 | Appm20 | 110/112 | *P2* | *P2-2 x Akaz* | no | . | 8 | 5 |
| L1 | Appm20 | 110/112 | *P2* | *P2-2 x Akaz* | no | . | 7 | 2 |
| L2 | Apcpm1 | 107/110 | *P1* | *P1-1 x Akaz-1* | no | . | 12 | 12 |
| L2 | Apcpm1 | 107/110 | *P1* | *P1-1 x Akaz-1* | no | . | 12 | 9 |
| L2 | Apcpm1 | 107/110 | *P1* | *P1-1 x Akaz-1* | no | . | 12 | 10 |
| L2 | Apcpm1 | 107/110 | *P1* | *P1-1 x Akaz-1* | no | . | 12 | 10 |
| L2 | Apcpm1 | 107/110 | *P1* | *P1-1 x Akaz-2* | no | . | 11 | 11 |
| L2 | Apcpm1 | 107/110 | *P1* | *P1-1 x Akaz-2* | no | . | 11 | 10 |
| L2 | Apcpm1 | 107/110 | *P1* | *P1-1 x Akaz-2* | no | . | 9 | 8 |
| L2 | Apcpm1 | 107/110 | *P1* | *P1-1 x Akaz-2* | NA | NA | 4 | 1 |
| L2 | Apcpm1 | 107/110 | *P1* | *P1-1 x Akaz-2* | NA | NA | 4 | 2 |
| L2 | Apcpm1 | 107/110 | *P1* | *P1-2 x Akaz* | no | . | 10 | 10 |
| L2 | Apcpm1 | 107/110 | *P1* | *P1-2 x Akaz* | no | . | 10 | 10 |
| L2 | Apcpm1 | 107/110 | *P1* | *P1-2 x Akaz* | no | . | 10 | 10 |
| L2 | Apcpm1 | 107/110 | *P1* | *P1-2 x Akaz* | no | . | 10 | 10 |
| L2 | Apcpm1 | 107/110 | *P1* | *P1-2 x Akaz* | no | . | 6 | 6 |
| L2 | Apcpm1 | 107/110 | *P2* | *P2-1 x Akaz* | no | . | 9 | 9 |
| L2 | Apcpm1 | 107/110 | *P2* | *P2-1 x Akaz* | no | . | 9 | 5 |
| L2 | Apcpm1 | 107/110 | *P2* | *P2-1 x Akaz* | no | . | 11 | 11 |
| L2 | Apcpm1 | 107/110 | *P2* | *P2-1 x Akaz* | no | . | 11 | 1 |
| L2 | Apcpm1 | 107/110 | *P2* | *P2-1 x Akaz* | no | . | 9 | 1 |
| L2 | Apcpm1 | 107/110 | *P2* | *P2-1 x Akaz* | no | . | 8 | 1 |
| L2 | Apcpm1 | 107/110 | *P2* | *P2-1 x Akaz* | no | . | 12 | 9 |
| L2 | Apcpm1 | 107/110 | *P2* | *P2-1 x Akaz* | no | . | 11 | 2 |
| L2 | Apcpm1 | 107/110 | *P2* | *P2-1 x Akaz* | no | . | 11 | 3 |
| L2 | Apcpm1 | 107/110 | *P2* | *P2-1 x Akaz* | no | . | 8 | 1 |
| L2 | Apcpm1 | 107/110 | *P2* | *P2-1 x Akaz* | no | . | 13 | 10 |
| L2 | Apcpm1 | 107/110 | *P2* | *P2-1 x Akaz* | no | . | 9 | 1 |
| L2 | Apcpm1 | 107/110 | *P2* | *P2-1 x Akaz* | no | . | 13 | 9 |
| L2 | Apcpm1 | 107/110 | *P2* | *P2-1 x Akaz* | no | . | 12 | 1 |
| L2 | Apcpm1 | 107/110 | *P2* | *P2-1 x Akaz* | no | . | 11 | 3 |
| L2 | Apcpm1 | 107/110 | *P2* | *P2-2 x Akaz* | no | . | 10 | 10 |
| L2 | Apcpm1 | 107/110 | *P2* | *P2-2 x Akaz* | no | . | 10 | 1 |
| L2 | Apcpm1 | 107/110 | *P2* | *P2-2 x Akaz* | no | . | 9 | 2 |
| L2 | Apcpm1 | 107/110 | *P2* | *P2-2 x Akaz* | no | . | 8 | 5 |
| L2 | Apcpm1 | 107/110 | *P2* | *P2-2 x Akaz* | no | . | 7 | 2 |
| L3 | Aupm16 | 128/132 | *P1* | *P1-1 x Akaz-1* | no | . | 12 | 12 |
| L3 | Aupm16 | 128/132 | *P1* | *P1-1 x Akaz-1* | no | . | 12 | 9 |
| L3 | Aupm16 | 128/132 | *P1* | *P1-1 x Akaz-1* | no | . | 12 | 10 |
| L3 | Aupm16 | 128/132 | *P1* | *P1-1 x Akaz-1* | no | . | 12 | 10 |
| L3 | Aupm16 | 128/132 | *P1* | *P1-1 x Akaz-2* | no | . | 11 | 11 |
| L3 | Aupm16 | 128/132 | *P1* | *P1-1 x Akaz-2* | no | . | 11 | 10 |
| L3 | Aupm16 | 128/132 | *P1* | *P1-1 x Akaz-2* | yes | 132/132 | 3 | 1 |
| L3 | Aupm16 | 128/132 | *P1* | *P1-1 x Akaz-2* | no | . | 4 | 3 |
| L3 | Aupm16 | 128/132 | *P1* | *P1-2 x Akaz* | no | . | 10 | 10 |
| L3 | Aupm16 | 128/132 | *P1* | *P1-2 x Akaz* | no | . | 10 | 10 |
| L3 | Aupm16 | 128/132 | *P1* | *P1-2 x Akaz* | no | . | 10 | 10 |
| L3 | Aupm16 | 128/132 | *P1* | *P1-2 x Akaz* | no | . | 10 | 10 |
| L3 | Aupm16 | 128/132 | *P1* | *P1-2 x Akaz* | no | . | 6 | 6 |
| L3 | Aupm16 | 128/132 | *P2* | *P2-2 x Akaz* | yes | 132/132 | 5 | 5 |
| L3 | Aupm16 | 128/132 | *P2* | *P2-2 x Akaz* | no | . | 8 | 5 |
| L3 | Aupm16 | 128/132 | *P2* | *P2-2 x Akaz* | no | . | 7 | 2 |
| L4 | Aupm21 | 108/113 | *P2* | *P2-2 x Akaz* | yes | 113/113 | 3 | 3 |
| L5 | AP02 | 230/233 | *P1* | *P1-1 x Akaz-1* | no | . | 12 | 12 |
| L5 | AP02 | 230/233 | *P1* | *P1-1 x Akaz-1* | no | . | 11 | 8 |
| L5 | AP02 | 230/233 | *P1* | *P1-1 x Akaz-1* | no | . | 12 | 10 |
| L5 | AP02 | 230/233 | *P1* | *P1-1 x Akaz-1* | no | . | 12 | 10 |
| L5 | AP02 | 230/233 | *P1* | *P1-1 x Akaz-2* | no | . | 11 | 11 |
| L5 | AP02 | 230/233 | *P1* | *P1-1 x Akaz-2* | no | . | 7 | 6 |
| L5 | AP02 | 230/233 | *P1* | *P1-1 x Akaz-2* | no | . | 9 | 8 |
| L5 | AP02 | 230/233 | *P1* | *P1-1 x Akaz-2* | NA | NA | 4 | 1 |
| L5 | AP02 | 230/233 | *P1* | *P1-1 x Akaz-2* | NA | NA | 4 | 2 |
| L5 | AP02 | 230/233 | *P1* | *P1-2 x Akaz* | no | . | 10 | 10 |
| L5 | AP02 | 230/233 | *P1* | *P1-2 x Akaz* | no | . | 10 | 10 |
| L5 | AP02 | 230/233 | *P1* | *P1-2 x Akaz* | yes | 233/233 | 8 | 8 |
| L5 | AP02 | 230/233 | *P1* | *P1-2 x Akaz* | no | . | 10 | 10 |
| L5 | AP02 | 230/233 | *P1* | *P1-2 x Akaz* | yes | 230/230 | 2 | 2 |
| L5 | AP02 | 230/242 | *P2* | *P2-1 x Akaz* | yes | 242/242 | 6 | 6 |
| L5 | AP02 | 230/242 | *P2* | *P2-1 x Akaz* | yes | 242/242 | 7 | 3 |
| L5 | AP02 | 230/242 | *P2* | *P2-1 x Akaz* | yes | 230/230 | 2 | 2 |
| L5 | AP02 | 221/230 | *P2* | *P2-2 x Akaz* | yes | 230/230 | 5 | 5 |
| L5 | AP02 | 221/230 | *P2* | *P2-2 x Akaz* | yes | 221/221 | 7 | 4 |
| L5 | AP02 | 221/230 | *P2* | *P2-2 x Akaz* | no | . | 7 | 2 |
| L6 | AP01 | 179/197 | *P2* | *P2-1 x Akaz* | no | . | 9 | 9 |
| L6 | AP01 | 179/197 | *P2* | *P2-1 x Akaz* | no | . | 9 | 5 |
| L6 | AP01 | 179/197 | *P2* | *P2-1 x Akaz* | no | . | 11 | 11 |
| L6 | AP01 | 179/197 | *P2* | *P2-1 x Akaz* | no | . | 11 | 1 |
| L6 | AP01 | 179/197 | *P2* | *P2-1 x Akaz* | no | . | 9 | 1 |
| L6 | AP01 | 179/197 | *P2* | *P2-1 x Akaz* | yes | 197/197 | 8 | 1 |
| L6 | AP01 | 179/197 | *P2* | *P2-1 x Akaz* | no | . | 13 | 10 |
| L6 | AP01 | 179/197 | *P2* | *P2-1 x Akaz* | no | . | 9 | 1 |
| L6 | AP01 | 179/197 | *P2* | *P2-1 x Akaz* | no | . | 13 | 9 |
| L6 | AP01 | 179/197 | *P2* | *P2-1 x Akaz* | no | . | 12 | 1 |
| L6 | AP01 | 179/197 | *P2* | *P2-1 x Akaz* | no | . | 11 | 3 |
| L6 | AP01 | 179/197 | *P2* | *P2-1 x Akaz* | no | . | 12 | 9 |
| L6 | AP01 | 179/197 | *P2* | *P2-1 x Akaz* | no | . | 11 | 2 |
| L6 | AP01 | 179/197 | *P2* | *P2-1 x Akaz* | no | . | 11 | 3 |
| L6 | AP01 | 179/197 | *P2* | *P2-1 x Akaz* | no | . | 8 | 1 |
| L6 | AP01 | 179/185 | *P2* | *P2-2 x Akaz* | yes | 185/185 | 5 | 5 |
| L6 | AP01 | 179/185 | *P2* | *P2-2 x Akaz* | no | . | 8 | 5 |
| L6 | AP01 | 179/185 | *P2* | *P2-2 x Akaz* | no | . | 7 | 2 |
| . | AP03 | 202/0 | *P2* | *P2-1 x Akaz* | yes | 0/0 | 1 | 1 |
| . | AP03 | 202/0 | *P2* | *P2-1 x Akaz* | yes | 0/0 | 3 | 3 |
| . | AP03 | 202/0 | *P2* | *P2-2 x Akaz* | yes | 0/0 | 5 | 5 |
| . | AP03 | 202/0 | *P2* | *P2-2 x Akaz* | NA | NA | 5 | 2 |

**Table S9.** Data for experiment 1. Marker name is the name of the markers as described in Muñoz et al. (2009) and Nougué et al. (2015). F1 genotype is the genotype of the first generation of each lineage. Cross refers to the cross as represented in Figure S1. LOH genotype is the genotype of the lineage after losing heterozygosity at a marker. True generations is the number of generation at which heterozygosity was lost or, alternatively, the generation until which the lineage was followed without LOH. Independent generations accounts for partial non-independence of some of the lineages due to sharing part of their ancestry.

| Cross | F1 female | Reproductive mode | Clutch in isolation | Clutch name | Clutch mode |
| --- | --- | --- | --- | --- | --- |
| *P2* x *Akaz* | P2-1 | asex | yes | P2-1_C1 | NA |
| *P2* x *Akaz* | P2-1 | asex | yes | P2-1_C2 | asex |
| *P2* x *Akaz* | P2-1 | asex | yes | P2-1_C3 | asex |
| *P2* x *Akaz* | P2-7 | mixed | yes | P2-7_C1 | sex |
| *P2* x *Akaz* | P2-7 | mixed | yes | P2-7_C2 | sex |
| *P2* x *Akaz* | P2-8 | sex | no | P2-8_C1 | sex |
| *P2* x *Akaz* | P2-8 | sex | no | P2-8_C2 | sex |
| *P2* x *Akaz* | P2-9 | NA | yes | P2-9_C1 | NA |
| *P2* x *Akaz* | P2-9 | NA | yes | P2-9_C2 | NA |
| *P2* x *Akaz* | P2-9 | NA | yes | P2-9_C3 | NA |
| *P2* x *Akaz* | P2-11 | mixed | yes | P2-11_C1 | sex |
| *P2* x *Akaz* | P2-19 | asex | no | P2-19_C1 | asex |
| *P2* x *Akaz* | P2-21 | asex | yes | P2-21_C1 | asex |
| *P2* x *Akaz* | P2-21 | asex | yes | P2-21_C2 | asex |
| *P2* x *Akaz* | P2-24 | sex | no | P2-24_C1 | sex |
| *P2* x *Akaz* | P2-24 | sex | no | P2-24_C2 | sex |
| *P2* x *Akaz* | P2-26 | sex | no | P2-26_C1 | sex |
| *P2* x *Akaz* | P2-27 | asex | no | P2-27_C1 | NA |
| *P2* x *Akaz* | P2-27 | asex | no | P2-27_C2 | NA |
| *P2* x *Akaz* | P2-27 | asex | no | P2-27_C3 | asex |
| *P2* x *Akaz* | P2-28 | asex | no | P2-28_C1 | asex |
| *P2* x *Akaz* | P2-28 | asex | no | P2-28_C2 | sex |
| *P2* x *Akaz* | P2-29 | asex | no | P2-29_C1 | asex |
| *P2* x *Akaz* | P2-29 | asex | no | P2-29_C2 | asex |
| *P2* x *Akaz* | P2-31 | sex | no | P2-31_C1 | sex |
| *P2* x *Akaz* | P2-31 | sex | no | P2-31_C2 | sex |
| *P2* x *Akaz* | P2-36 | asex | yes | P2-36_C1 | asex |
| *P2* x *Akaz* | P2-39 | asex | no | P2-39_C2 | NA |
| *P2* x *Akaz* | P2-40 | mixed | no | P2-40_C1 | sex |
| *P2* x *Akaz* | P2-40 | mixed | no | P2-40_C2 | asex |
| *P2* x *Akaz* | P2-42 | mixed | yes | P2-42_C1 | sex |
| *P2* x *Akaz* | P2-45 | asex | yes | P2-45_C1 | asex |
| *P2* x *Akaz* | P2-45 | asex | yes | P2-45_C2 | NA |
| *P2* x *Akaz* | P2-45 | asex | yes | P2-45_C3 | asex |
| *P2* x *Akaz* | P2-47 | asex | yes | P2-47_C1 | NA |
| *P2* x *Akaz* | P2-47 | asex | yes | P2-47_C2 | NA |
| *P2* x *Akaz* | P2-47 | asex | yes | P2-47_C3 | asex |
| *P2* x *Akaz* | P2-51 | asex | yes | P2-51_C1 | asex |
| *P2* x *Akaz* | P2-55 | NA | no | P2-55_C1 | NA |
| *P2* x *Akaz* | P2-57 | asex | no | P2-57_C1 | asex |
| *P2* x *Akaz* | P2-58 | mixed | no | P2-58_C1 | sex |
| *P2* x *Akaz* | P2-58 | mixed | no | P2-58_C2 | asex |
| *P2* x *Akaz* | P2-58 | mixed | no | P2-58_C3 | sex |
| *P2* x *Akaz* | P2-58 | mixed | no | P2-58_C4 | sex |
| *P2* x *Akaz* | P2-61 | asex | yes | P2-61_C1 | asex |
| *P2* x *Akaz* | P2-62 | asex | yes | P2-62_C1 | asex |
| *P2* x *Akaz* | P2-62 | asex | yes | P2-62_C2 | asex |
| *P2* x *Akaz* | P2-62 | asex | yes | P2-62_C3 | NA |
| *P2* x *Akaz* | P2-63 | asex | yes | P2-63_C1 | NA |
| *P2* x *Akaz* | P2-63 | asex | yes | P2-63_C2 | asex |
| *P2* x *Akaz* | P2-64 | asex | yes | P2-64_C1 | asex |
| *P2* x *Akaz* | P2-65 | asex | no | P2-65_C1 | asex |
| *P2* x *Akaz* | P2-66 | mixed | yes | P2-66_C1 | sex |
| *P2* x *Akaz* | P2-66 | sex | yes | P2-66_C2 | NA |
| *P2* x *Akaz* | P2-66 | sex | yes | P2-66_C3 | NA |
| *P2* x *Akaz* | P2-68 | asex | no | P2-68_C1 | asex |
| *P2* x *Akaz* | P2-68 | asex | no | P2-68_C2 | asex |
| *P2* x *Akaz* | P2-68 | asex | no | P2-68_C3 | asex |
| *P2* x *Akaz* | P2-71 | sex | no | P2-71_C1 | sex |
| *P2* x *Akaz* | P2-72 | mixed | yes | P2-72_C1 | sex |
| *P2* x *Akaz* | P2-72 | sex | yes | P2-72_C2 | NA |
| *P2* x *Akaz* | P2-73 | asex | yes | P2-73_C1 | asex |
| *P2* x *Akaz* | P2-73 | asex | yes | P2-73_C2 | asex |
| *P2* x *Akaz* | P2-73 | asex | yes | P2-73_C3 | NA |
| *P2* x *Akaz* | P2-74 | asex | yes | P2-74_C1 | asex |
| *P2* x *Akaz* | P2-74 | asex | yes | P2-74_C2 | asex |
| *P2* x *Akaz* | P2-74 | asex | yes | P2-74_C3 | asex |
| *P2* x *Akaz* | P2-75 | asex | yes | P2-75_C1 | asex |
| *P2* x *Akaz* | P2-75 | asex | yes | P2-75_C2 | NA |
| *P2* x *Akaz* | P2-82 | asex | yes | P2-82_C1 | asex |
| *P2* x *Akaz* | P2-82 | asex | yes | P2-82_C2 | asex |
| *P2* x *Akaz* | P2-82 | asex | yes | P2-82_C3 | asex |
| *P1* x *Akaz* | P1-2 | asex | yes | P1-2_C1 | asex |
| *P1* x *Akaz* | P1-2 | asex | yes | P1-2_C2 | asex |
| *P1* x *Akaz* | P1-2 | asex | yes | P1-2_C3 | asex |
| *P1* x *Akaz* | P1-2 | asex | yes | P1-2_C4 | asex |
| *P1* x *Akaz* | P1-2 | asex | yes | P1-2_C5 | asex |
| *P1* x *Akaz* | P1-3 | asex | yes | P1-3_C1 | asex |
| *P1* x *Akaz* | P1-3 | asex | yes | P1-3_C2 | asex |
| *P1* x *Akaz* | P1-9 | asex | yes | P1-9_C1 | asex |
| *P1* x *Akaz* | P1-9 | asex | yes | P1-9_C2 | asex |
| *P1* x *Akaz* | P1-9 | asex | yes | P1-9_C3 | asex |
| *P1* x *Akaz* | P1-11 | mixed | yes | P1-11_C1 | asex |
| *P1* x *Akaz* | P1-11 | mixed | yes | P1-11_C2 | sex |
| *P1* x *Akaz* | P1-14 | asex | yes | P1-14_C1 | asex |
| *P1* x *Akaz* | P1-14 | asex | yes | P1-14_C2 | NA |
| *P1* x *Akaz* | P1-14 | asex | yes | P1-14_C3 | NA |
| *P1* x *Akaz* | P1-14 | asex | yes | P1-14_C4 | NA |
| *P1* x *Akaz* | P1-14 | asex | yes | P1-14_C5 | NA |
| *P1* x *Akaz* | P1-15 | mixed | yes | P1-15_C1 | sex |
| *P1* x *Akaz* | P1-15 | mixed | yes | P1-15_C2 | sex |
| *P1* x *Akaz* | P1-15 | mixed | yes | P1-15_C3 | sex |
| *P1* x *Akaz* | P1-18 | asex | yes | P1-18_C1 | NA |
| *P1* x *Akaz* | P1-18 | asex | yes | P1-18_C2 | asex |
| *P1* x *Akaz* | P1-18 | asex | yes | P1-18_C3 | asex |
| *P1* x *Akaz* | P1-18 | asex | yes | P1-18_C4 | asex |
| *P1* x *Akaz* | P1-19 | asex | yes | P1-19_C1 | asex |
| *P1* x *Akaz* | P1-19 | asex | yes | P1-19_C2 | asex |
| *P1* x *Akaz* | P1-19 | asex | yes | P1-19_C3 | asex |
| *P1* x *Akaz* | P1-20 | asex | yes | P1-20_C1 | asex |
| *P1* x *Akaz* | P1-20 | asex | yes | P1-20_C2 | asex |
| *P1* x *Akaz* | P1-20 | asex | yes | P1-20_C3 | asex |
| *P1* x *Akaz* | P1-20 | asex | yes | P1-20_C4 | asex |
| *P1* x *Akaz* | P1-21 | mixed | yes | P1-21_C1 | asex |
| *P1* x *Akaz* | P1-21 | mixed | yes | P1-21_C2 | asex |
| *P1* x *Akaz* | P1-21 | mixed | yes | P1-21_C3 | asex |
| *P1* x *Akaz* | P1-21 | mixed | yes | P1-21_C4 | asex |
| *P1* x *Akaz* | P1-21 | mixed | yes | P1-21_C5 | sex |
| *P1* x *Akaz* | P1-23 | asex | yes | P1-23_C1 | asex |
| *P1* x *Akaz* | P1-23 | asex | yes | P1-23_C2 | asex |
| *P1* x *Akaz* | P1-24 | mixed | yes | P1-24_C1 | asex |
| *P1* x *Akaz* | P1-24 | mixed | yes | P1-24_C2 | asex |
| *P1* x *Akaz* | P1-24 | mixed | yes | P1-24_C3 | sex |
| *P1* x *Akaz* | P1-24 | mixed | yes | P1-24_C4 | asex |
| *P1* x *Akaz* | P1-26 | mixed | no | P1-26_C1 | asex |
| *P1* x *Akaz* | P1-26 | mixed | no | P1-26_C2 | asex |
| *P1* x *Akaz* | P1-26 | mixed | no | P1-26_C3 | sex |
| *P1* x *Akaz* | P1-29 | asex | no | P1-29_C1 | asex |
| *P1* x *Akaz* | P1-29 | asex | no | P1-29_C2 | asex |
| *P1* x *Akaz* | P1-29 | asex | no | P1-29_C3 | asex |
| *P1* x *Akaz* | P1-29 | asex | no | P1-29_C4 | asex |
| *P1* x *Akaz* | P1-35 | asex | yes | P1-35_C1 | asex |
| *P1* x *Akaz* | P1-35 | asex | yes | P1-35_C2 | asex |
| *P1* x *Akaz* | P1-35 | asex | yes | P1-35_C3 | asex |
| *P1* x *Akaz* | P1-35 | asex | yes | P1-35_C4 | asex |
| *P1* x *Akaz* | P1-36 | asex | yes | P1-36_C2 | asex |
| *P1* x *Akaz* | P1-36 | asex | yes | P1-36_C3 | asex |
| *P1* x *Akaz* | P1-36 | asex | yes | P1-36_C4 | asex |
| *P1* x *Akaz* | P1-36 | asex | yes | P1-36_C5 | asex |
| *P1* x *Akaz* | P1-37 | asex | yes | P1-37_C2 | asex |
| *P1* x *Akaz* | P1-37 | asex | yes | P1-37_C3 | asex |
| *P1* x *Akaz* | P1-37 | asex | yes | P1-37_C4 | asex |
| *P1* x *Akaz* | P1-38 | asex | yes | P1-38_C1 | asex |
| *P1* x *Akaz* | P1-38 | asex | yes | P1-38_C2 | asex |
| *P1* x *Akaz* | P1-39 | asex | yes | P1-39_C1 | asex |
| *P1* x *Akaz* | P1-39 | asex | yes | P1-39_C2 | asex |
| *P1* x *Akaz* | P1-39 | asex | yes | P1-39_C3 | asex |
| *P1* x *Akaz* | P1-39 | asex | yes | P1-39_C4 | asex |
| *P1* x *Akaz* | P1-42 | asex | yes | P1-42_C1 | asex |
| *P1* x *Akaz* | P1-42 | asex | yes | P1-42_C2 | asex |
| *P1* x *Akaz* | P1-42 | asex | yes | P1-42_C3 | asex |
| *P1* x *Akaz* | P1-42 | asex | yes | P1-42_C4 | asex |
| *P1* x *Akaz* | P1-43 | asex | yes | P1-43_C1 | asex |
| *P1* x *Akaz* | P1-43 | asex | yes | P1-43_C2 | asex |
| *P1* x *Akaz* | P1-43 | asex | yes | P1-43_C3 | asex |
| *P1* x *Akaz* | P1-44 | mixed | yes | P1-44_C1 | asex |
| *P1* x *Akaz* | P1-44 | mixed | yes | P1-44_C2 | sex |
| *P1* x *Akaz* | P1-44 | mixed | yes | P1-44_C3 | asex |
| *P1* x *Akaz* | P1-44 | mixed | yes | P1-44_C4 | asex |
| *P1* x *Akaz* | P1-45 | asex | yes | P1-45_C1 | NA |
| *P1* x *Akaz* | P1-45 | asex | yes | P1-45_C2 | NA |
| *P1* x *Akaz* | P1-45 | asex | yes | P1-45_C3 | NA |
| *P1* x *Akaz* | P1-46 | asex | yes | P1-46_C1 | asex |
| *P1* x *Akaz* | P1-46 | asex | yes | P1-46_C2 | asex |
| *P1* x *Akaz* | P1-46 | asex | yes | P1-46_C3 | asex |
| *P1* x *Akaz* | P1-47 | mixed | yes | P1-47_C1 | NA |
| *P1* x *Akaz* | P1-47 | mixed | yes | P1-47_C2 | NA |
| *P1* x *Akaz* | P1-47 | mixed | yes | P1-47_C3 | sex |
| *P1* x *Akaz* | P1-47 | mixed | yes | P1-47_C4 | sex |
| *P1* x *Akaz* | P1-47 | mixed | yes | P1-47_C5 | NA |
| *P1* x *Akaz* | P1-49 | NA | no | P1-49_C1 | NA |
| *P1* x *Akaz* | P1-49 | NA | no | P1-49_C2 | NA |
| *P1* x *Akaz* | P1-49 | NA | no | P1-49_C3 | NA |
| *P1* x *Akaz* | P1-50 | asex | yes | P1-50_C1 | asex |
| *P1* x *Akaz* | P1-51 | asex | yes | P1-51_C1 | asex |
| *P1* x *Akaz* | P1-51 | asex | yes | P1-51_C2 | asex |
| *P1* x *Akaz* | P1-51 | asex | yes | P1-51_C4 | asex |

**Figure S10.** Data for experiment 2. F1 female is the name of the F1 female tested. Clutch in isolation indicates whether the female reproduced asexually while isolated (period 1). Clutch name is the unique name of each clutch produced during pairing with an *Akaz* male (period 2). Clutch mode is the reproductive mode identified for each clutch.
